# High-throughput detection of mutations responsible for childhood hearing loss using resequencing microarrays

**DOI:** 10.1186/1472-6750-10-10

**Published:** 2010-02-10

**Authors:** Prachi Kothiyal, Stephanie Cox, Jonathan Ebert, Ammar Husami, Margaret A Kenna, John H Greinwald, Bruce J Aronow, Heidi L Rehm

**Affiliations:** 1 Biomedical Informatics, Cincinnati Children's Hospital Medical Center, Cincinnati, Ohio 45229, USA; 2 Department of Biomedical Engineering, University of Cincinnati, Cincinnati, Ohio 45221, USA; 3 Partners Healthcare Center for Personalized Genetic Medicine, Boston, Massachusetts 02115, USA; 4 Ear and Hearing Center, Cincinnati Children's Hospital Medical Center, Cincinnati, Ohio 45229, USA; 5 Department of Otolaryngology and Communication Enhancement, Children's Hospital Boston, Massachusetts 02115, USA; 6 Department of Otology and Laryngology, Harvard Medical School, Boston, Massachusetts 02115, USA; 7 Department of Otolaryngology, Head and Neck Surgery, University of Cincinnati College of Medicine, Cincinnati, Ohio 45221, USA; 8 Department of Pathology, Brigham & Women's Hospital and Harvard Medical School, Boston, Massachusetts 02115, USA

## Abstract

**Background:**

Despite current knowledge of mutations in 45 genes that can cause nonsyndromic sensorineural hearing loss (SNHL), no unified clinical test has been developed that can comprehensively detect mutations in multiple genes. We therefore designed Affymetrix resequencing microarrays capable of resequencing 13 genes mutated in SNHL (*GJB2, GJB6, CDH23, KCNE1, KCNQ1, MYO7A, OTOF, PDS, MYO6, SLC26A5, TMIE, TMPRSS3, USH1C*). We present results from hearing loss arrays developed in two different research facilities and highlight some of the approaches we adopted to enhance the applicability of resequencing arrays in a clinical setting.

**Results:**

We leveraged sequence and intensity pattern features responsible for diminished coverage and accuracy and developed a novel algorithm, sPROFILER, which resolved >80% of no-calls from GSEQ and allowed 99.6% (range: 99.2-99.8%) of sequence to be called, while maintaining overall accuracy at >99.8% based upon dideoxy sequencing comparison.

**Conclusions:**

Together, these findings provide insight into critical issues for disease-centered resequencing protocols suitable for clinical application and support the use of array-based resequencing technology as a valuable molecular diagnostic tool for pediatric SNHL and other genetic diseases with substantial genetic heterogeneity.

## Background

The medical evaluation of sensorineural hearing loss (SNHL) involves a combination of non-genetic laboratory and radiographic tests. The former provide little diagnostic or prognostic information [1]. Radiographic evaluations are helpful in diagnosing temporal bone anomalies, but are expensive and require sedation or general anaesthesia in children [2]. Additionally, these tests are time-consuming and stressful for the child and family. Most recently, genetic testing of the *GJB2 *gene has been added to the diagnostic evaluation. Mutations in this gene account for about 20% of children with nonsyndromic SNHL [3]. Recent data has demonstrated the utility of *GJB2 *analysis in determining prognosis, the best intervention, and recurrence risks to future children and other family members [4,5]. Genetic testing can also predict the absence or onset of a syndrome for which the other clinical problems may not be present at birth or early childhood (e.g. adolescent-onset blindness in Usher syndrome). When the genetic etiology can be determined in a large cohort of patients, it will provide a better understanding of the genotype-phenotype correlations that exist for each of the genes examined, which could direct specific therapeutic interventions.

Beyond *GJB2*, it is uncertain what genetic mutations are the next most prevalent in patients with hearing loss. Other genes thought to play a significant role in childhood hearing loss include *SLC26A4 *[6], Usher type 1 genes (e.g. *MYO7A, CDH23*) [7,8] and *OTOF *[9], though no studies have looked at many genes to appreciate their relative contributions. The identification of over 45 genes causative for SNHL now makes it imperative to develop a high-throughput resequencing assay. Such a technology would allow for a more comprehensive and therefore higher yield diagnostic evaluation of the etiology of hearing loss in patients. Current technology allows for widespread screening of only the most common genes related to SNHL (i.e. *GJB2*).

Recent advances in molecular microarray technology have made it feasible to rapidly screen DNA samples for thousands of possible genetic mutations [10-14]. The advantages of microarray-based screening include its accuracy, simplicity, efficiency and cost-effectiveness when employed on a large scale. However, call rates among different microarray designs may vary considerably [11,15,16]. While computational methods for reduction in false positives due to systematic effects have previously been proposed [17], inadequate call coverage is also a considerable limitation of the resequencing array-based approach [13,15].

The efficiency of sequence-specific hybridization is dependent on the properties of the probe and target sequences [18]. High GC-content, presence of a nearby SNP and cross-hybridizing sequences are known to affect base-calling, thus limiting the capacity of resequencing microarrays. It has been shown that C-rich probes perform better than the complementary G-rich probes [19]. Additionally, it has been reported that nearly 80% of no-calls can be resolved by visual inspection of the intensities as one of the strands provides a clear signature for these positions [13]. However, there are no existing computational approaches that leverage such sequence-specific characteristics in an attempt to resolve GSEQ no-calls that have a distinct signature on one strand but are still ruled no-call due to improper hybridization on the complementary strand.

GSEQ is known to produce very few false negatives, thus providing a highly sensitive test. However, follow-up dideoxy sequencing for resolution of no-calls leads to an additional variable cost, a factor which needs to be carefully considered for clinical application of the technology. To this effect, we propose a novel algorithm for resolution of no-calls from GSEQ. It should be noted that the algorithm is not designed to be an alternative to GSEQ. Instead, it provides an optional step for salvaging unresolved bases from GSEQ before initiating confirmatory dideoxy sequencing.

Our work focuses on evaluating the effectiveness of resequencing arrays as a tool for variant detection and discusses the impact on base-calling of adopting additional computational algorithms and laboratory protocols. This study presents the results from hearing loss arrays developed in two different research facilities and highlights some of the approaches we adopted to enhance the applicability of the arrays in a clinical setting.

## Results

### Overall array performance

The Harvard array contained 8 genes (see **Methods**). Performance characteristics were determined from data analyzed for a set of 26 arrays run after protocol optimization (Table 1). The average base call rate across the 26 arrays (654,862 bases) was 96.9% using Affymetrix GDAS 2.0. We confirmed every variant call with dideoxy sequencing to determine the false positive rate. On average, about 57 variants were called per array but only 28% were true variants and the rest were false positives. Dideoxy sequencing of 352,618 bases across 14 arrays was performed and the data compared to array calls. Factoring in false negatives and false positives, we obtained an average base call accuracy of 99.82% across the 14 arrays.

**Table 1:** Overall array performance with and without application of sPROFILER to GDAS/GSEQ base calls.

	Harvard arrays with GDAS*	Harvard arrays with GDAS/sPROFILER*	Cincinnati arrays with GSEQ	Cincinnati arrays with GSEQ/sPROFILER
**Number of arrays**	26	26	13	13
**Bases per array**	25187	25187	26292	26292
**Array call rate**^**A**^	96.9%	99.6%	97.9%	99.6%
**Call accuracy**^**B**^	99.82%	99.84%	99.83%	99.88%
**Total false positive rate**^**C**^	0.18% (41)	0.15% (38)	0.16% (42)	0.11% (30)
**Total false negative rate**^**D**^	0.0016% (0.4)	0.0031% (0.9)	0.0009% (0.2)	0.0020% (0.6)
**Variant false positive rate**^**E**^	72.6% (41/57)	71.6% (38/51)	77.7% (42/54)	69.7% (30/43)
**Variant false negative rate**^**F**^	2.4% (0.4/16)	4.5% (0.9/16)	1.3% (0.2/15)	3.0% (0.6/15)
**No-calls**^**G**^	781	101	563	103
**No. of exons to be sequenced^**H**^/Total no. of exons on array**	153/196	52/196	150/180	68/180

Percentages are obtained by averaging individual percent values over all arrays.

A: Bases called/total bases on array

B: Correct calls/total calls

C: Wild-type bases incorrectly identified as variants/total calls * 100% (average raw # per array)

D: True variants incorrectly called wild-type/total calls * 100% (average raw # per array)

E: Wild-type bases incorrectly identified as variants/total variant calls * 100%

F: True variants incorrectly called wild-type/total true variants * 100%

G: Average number of bases not called per array

H: Number of exons that need follow-up sequencing to interrogate no-calls or variant calls

*: 14 Harvard arrays with full dideoxy sequencing results were used for determination of false negatives and overall accuracy. However, no-calls and variant calls across all 26 Harvard arrays were used for call rates and false positive rates.

**Figure 1:**
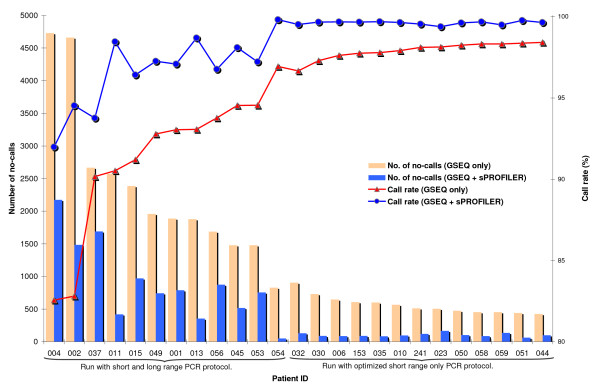
**Improvement in array call rates with protocol optimization and application of sPROFILER to GSEQ calls**. (data shown for Cincinnati arrays). Data is separated into two categories based upon protocol (short and long range PCR vs. short range only PCR) and then arranged in ascending order of GSEQ call rates.

**Figure 2:**
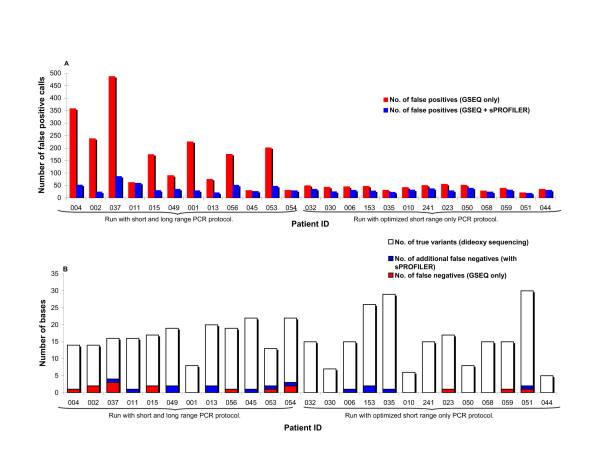
**Performance improvement with protocol optimization; array sensitivity and specificity with application of sPROFILER to GSEQ calls**. (data shown for Cincinnati arrays). Data is arranged in the same patient ID order as figure 1. **(a) **False positive calls with and without protocol optimization/sPROFILER. No-calls and positive calls were processed for the first 12 chips (short and long range PCR protocol) while only no-calls were processed for the remaining 13 chips (short range only PCR protocol). No-calls were converted to wild-type, left as no-call, or were assigned a variant call. Chips that were analyzed only for no-calls may show an increase in false positive rate due to conversion of a fraction of no-calls to variant calls, some of which are not true variants. **(b) **False negative calls with and without protocol optimization/sPROFILER represented as a portion of total true variants.

The Cincinnati array also contained 8 genes, 3 of which were common to the Harvard array (see **Methods**). We ran 12 arrays in the pilot batch and characterized array performance. Base call rates for these 12 arrays (315,504 bases) ranged from 82.5% to 96.9% with an average call rate of 91.3% using GSEQ 4.0 (Figures 1 &2). Dideoxy sequencing was performed for 296,296 of the bases and comparison of this data with array calls gave a call accuracy of 99.23% with nearly 180 false positives per array.

### Improved array performance with protocol optimization

When we compared data from Cincinnati and Harvard arrays, the number of no-calls and false positives from the former were found to be higher. Early data from the Harvard arrays had shown that call rate worsened when the fragmentation was incomplete and that the bases most affected were those within the long range PCR fragments. In addition, the reproducibility of the quantity of product from long range PCR was less. Based upon these two factors, the target amplification process for Cincinnati arrays was modified. Instead of using a combination of long and short range PCR (54 fragments ranging from 434 to 13,282 bases in length), as was employed in the pilot batch, all long range PCRs were converted to short range resulting in 180 fragments ranging from 315 to 980 bases in length. The impact of using shorter PCR products was evaluated by comparing array data across the two protocols (Figures 1 &2). A total of 13 arrays (341,796 bases) were run with the optimized "short range PCR only" protocol and an average call rate of 97.9% (range 96.7% to 98.4%) was obtained with GSEQ (Table 1). Dideoxy sequencing was performed for 336,171 of the bases and comparison of this data with array calls gave an array call accuracy of 99.83%. The average number of false positives dropped from 180 to 42 per array. The modified protocol with shorter PCR products was then adopted for subsequent arrays.

### Detection of insertions and deletions

Through dideoxy sequencing, we identified four cases with at least one insertion or deletion (35delG/35delG, 35delG/167delT, and M1V/167delT in *GJB2 *and homozygous 1180_1187del8ins(β-sat) in *TMPRSS3*), of which 2 had been previously reported and therefore had probes tiled on the array for their detection (*GJB2 *35delG and 167delT) (Additional file 1). We analyzed array data to look for no-calls or variant calls in the vicinity of the indel sites but did not observe any such patterns for the 35delG and 167delT alleles. It should be noted that the local high GC-content surrounding the 35delG (stretch of 5 Gs; probe GC 56%) and the 167delT (stretch of 4 Cs; probe GC 64%) would be expected to make detection extremely difficult. On the other hand, there was a continuous stretch of 13 no-calls and a variant call spanning the deleted bases of the *TMPRSS3 *gene that led to the detection of the mutation. We also analyzed raw feature intensities within fragments to see if indels cause degradation in intensities surrounding the variant site. We observed lower peak intensities surrounding the *TMPRSS3 *mutation but did not find such evidence for either of the single base *GJB2 *deletions (data not shown). GDAS/GSEQ are currently not designed for identifying indels so their low detection rate was an expected observation.

### Differential impact of high C-content and G-content on probe performance

**Figure 3:**
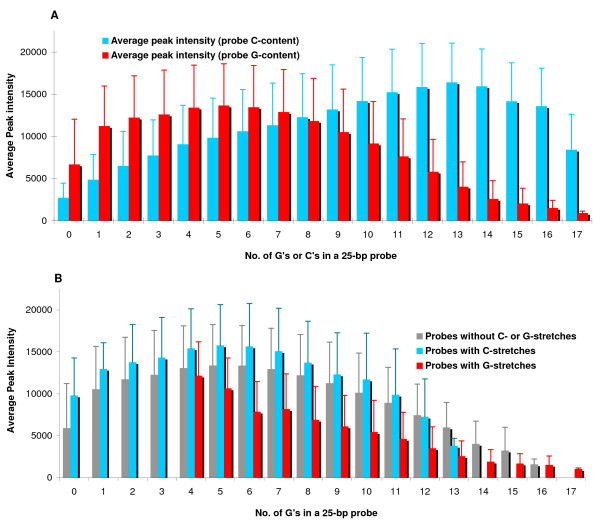
**Differential impact of high probe G-content and C-content on probe performance; G-richness of a probe has a more severe impact on hybridization intensity than C-richness and G-stretches degrade peak intensity**. **(a) **Peak feature intensity versus probe G-content and C-content. **(b) **Peak feature intensity for probes with same G-content grouped based on presence of G-stretch, C-stretch and no continuous stretches. Error bars represent one standard deviation.

In agreement with previously reported findings [19,20], we observed that an increase in probe G- and C-contents have differential impact on performance. We used complementary feature quartets to determine intensities associated with the C- or G-content of a probe. Intensity characteristics varied differently with respect to an increase in C- or G-content and average peak intensity was affected more severely by a high G-content than by an equally high C-content (Figure 3a). In order to assess if our data demonstrated previously reported debilitating effect of stretches of Gs (G-stacks) on probe performance [21], we compared hybridization intensities among probes with the same G-content grouped based on the presence or absence of G-stretches (≥ 4 Gs in a continuous stretch). For the same G-content, probes with G-stretches produced lower peak intensities than probes with C-stretches or without any stretches of Gs or Cs (Figure 3b).

### Overall array performance with sPROFILER

sPROFILER was only used on no-calls from GDAS/GSEQ. Examined bases were called wild-type based on single-strand evidence or were left as no-calls depending upon the feature intensity profile. Base calls were subsequently compared against GSEQ and dideoxy sequencing calls. Table 1 and Figures 1 &2 provide detailed comparison of call rates, number of false positives and false negatives before and after analyzing GSEQ calls with sPROFILER. For Cincinnati arrays run with short and long range PCR fragments, the average call rate increased to 96.7% (99.82% call accuracy). The average number of no-calls dropped from 2350 to 902 per array and the number of false positives dropped from 180 to 44 bases per array. Improvement was obtained at the cost of incorrectly assigning an additional 0.38 true variants per array as wild-type. For the optmized Cincinnati arrays, average call rate increased to 99.6% (range 99.5% to 99.8%) with 99.88% call accuracy and number of no-calls dropped from 563 to 103 (Table 1). The number of false positives dropped from 42 to 30 per array with the application of filters to screen variant calls based on low quality scores and the number of no-calls and variant calls in the vicinity. Improvement was achieved at the cost of an additional 0.4 false negative per array. We also calculated the number of exons per array that would need to be interrogated with dideoxy sequencing in order to resolve no-calls and confirm positive calls. After processing GSEQ calls with sPROFILER, the average number of exons to be sequenced in order to clarify no-calls and confirm variants dropped from 150 (range 132 to 165) to 68 (range 52 to 82) per array.

When using sPROFILER on the Harvard arrays, the average call rate increased from 96.9% to 99.6% and number of no-calls dropped from 781 to 101 per array (Table 1). False positive calls could be reduced from 41 to 38 per array with the filters for false positive calls. An additional 0.4 variants were falsely called wild-type using the algorithm. Because all no-calls are followed up by dideoxy sequencing, the implementation of the sPROFILER resulted in substantially fewer exons (average of 153 down to 52) needing follow-up.

### Clinical sensitivity of arrays

A detailed list of all variants and associated frequencies in the patient cohorts and control populations is included in Additional file 1. A total of 411 true variants were detected across 26 Harvard arrays, out of which, 10 were called wildtype by GDAS (Table 2). Interestingly, the 10 missed variants represent a single common SNP (*MYO7A *4755T>C) seen in 10 different arrays. As such, it appears that this single substitution may be problematic. The basis of the poor sensitivity for this variant did not appear to be overall high GC-content, which was 52%, nor a local G/C stretch (only one adjacent base was a G). Of the 411 variants, 44 represent rare variants, many of potential clinical significance. None of these were missed by the array, although 12 were assigned no-calls. Of the 192 true variants identified across the 13 optimized Cincinnati arrays, 3 synonymous variants (*CDH23 *2761C>T, *KCNQ1 *1185C>T, *MYO7A *4831C>T) were called wild-type by GSEQ (Table 2). The *CDH23 *and *MYO7A *variants were found to lie within a G/C stretch with probe GC-contents of 68% and 52% respectively but the *KCNQ1 *variant was not associated with high GC-content or a G/C stretch.

**Table 2:** Breakdown of validated variant calls across Cincinnati and Harvard arrays

	Harvard (26 arrays)		Cincinnati (13 arrays)	
	Total	Per array average	Total	Per array average
**Total variants**	411	16	192	15
**Total unique variants**	50	16	61	15
				
**Common variants detected**				
**Correctly called**	292	11	141	11
**No call**	57	2.2	23	1.8
**Missed call (het vs. hom)**	8	0.3	10	0.8
**Called wild-type**	10^A^	0.4	0	0
				
**Rare variants detected (1 case)**				
**Correctly called**	30	1.2	10	0.8
**No call**	12	0.5	5	0.4
**Missed call (het vs. hom)**	2	0.1	0	0
**Called wild-type**	0	0	3^B^	0.2

A: All 10 wild-type calls (false negatives) were due to a single repeatedly miscalled common benign variant in *MYO7A *(4755T>C; S1585S).

B: False negatives were from *CDH23 *(2761C>T, L921L), *MYO7A *(4831C>T, L1611L) and *KCNQ1 *(1185C>T, F395F)

After excluding synonymous and/or common variants, 18/37 patients examined by the Cincinnati array and 9/24 patients examined on the Harvard array had at least one variant of potential or likely clinical significance (excludes two cases known to have *GJB2 *biallelic mutations). See Additional file 1 for a full list of all variants found on a per patient basis. To further assess the likelihood that each variant may be disease-causing, efforts are currently underway to examine these variants in further control studies and using in silico algorithms to predict protein impact. After these studies are completed, variants with potential clinical significance will be confirmed in a CLIA environment with results returned to patients under an IRB approved protocol.

## Discussion

Serial molecular techniques (e.g., direct sequencing, single-strand conformation analysis, denaturing gradient gel electrophoresis and denaturing high-performance liquid chromatography) have been employed for detection of mutations associated with disorders showing high genetic and allelic heterogeneity but they can be laborious requiring high turnaround times and show little difference in their direct costs per base, which are high [12]. Conventional serial methods can be especially ineffective for screening large genes without definite hot spots for disease-associated mutations [10]. Although new advancements in next generation sequencing will soon replace all large scale sequencing platforms, these technologies are still too costly for medium size applications of targeted disease sequencing. High-density oligonucleotide microarrays provide an efficient and economically competitive method for genetic screening of heterogeneous disorders by allowing parallel resequencing of multiple genes in a single experiment. Since the first study reporting detection of known genomic variants using oligonucleotide arrays [22], several others have been published describing the principles of resequencing array technology [18,20,23,24] and its application for genotyping in prokaryotes and eukaryotes [10-14,25-27].

We developed two resequencing microarrays containing 13 unique genes implicated in nonsyndromic SNHL. Array base calls were compared to dideoxy sequencing to determine accuracy. Through optimization of protocols and data analysis methods, similar high-quality performance measures could be achieved for microarrays developed at two independent research facilities and containing different sets of genes.

The critical performance characteristics we attempted to understand and optimize are call rate, sensitivity and specificity. Affymetrix GSEQ is an upgraded version of the GDAS base-calling software and offers some additional features as described in the GSEQ technical datasheet http://www.affymetrix.com/support/technical/datasheets/gseq_datasheet.pdf. However, they both employ a base-calling algorithm built upon the adaptive background genotyping-calling scheme (ABACUS) developed by Cutler and colleagues [20]. GDAS and GSEQ produce few false negatives because these algorithms are conservative in making wild-type calls. False positives or no-calls do not represent a lack of test sensitivity when they are followed up by dideoxy sequencing; however, they compromise the cost-effectiveness of the technology if a large amount of sequencing is required. In our hands, the cost reduction is roughly a 25%-50% reduction compared to traditional capillary sequencing when thorough follow-up is employed to resolve all variant calls and rare no-calls. The exact reduction depends on the degree of multiplexing employed in the up-front PCR step and the amount of follow-up sequencing that is needed. The latter factor is unique to each test depending on the sequences included, PCR robustness, the amount of DNA variation in the regions tested and degree of bioinformatics and test optimization that has been achieved.

It has previously been suggested that large PCR amplicons do not hybridize efficiently to immobilized probes possibly due to steric constraints on the approach of the target DNA [28] and this finding has been taken into consideration during design of nucleic acid amplification strategies [29]. Optimizing the target amplification process to include only short range PCR improved the overall array performance in the Cincinnati arrays, thus providing further evidence for the relationship between PCR amplicon length and hybridization efficiency. It should be noted that the data generated by the Harvard arrays was based upon a combination of short range and long range PCR. However, the Harvard group has also discontinued use of long range PCR in subsequently developed array-based sequencing tests. This is because the efforts needed to continually optimize the fragmentation of long range PCR fragments and the additional limitations caused by diminished DNA quantity and variable amplification efficiency in long range PCR do not outweigh the benefits. For most nuclear genes with dispersed exons, only one to a handful of exons can be combined into a long range PCR reaction limiting the efficiency gained by this approach. In contrast, amplification of long stretches of contiguous interrogated DNA, such as that present in the mitochondrial genome, enables the highest efficiency savings for long-range PCR approaches.

Average call rates of 96.9% and 97.7% for all of Harvard arrays and the optimized Cincinnati arrays respectively were achieved using GDAS/GSEQ. Previous resequencing array-based studies [10-14] have reported call rates ranging from 93.5% to 98% with GDAS/GSEQ. While our call rates are within the high end of reported ranges, a large percentage (~80%) of the tiled exons required sequencing to follow-up on ambiguous calls, representing a limitation to clinical application of the technology under the current methodologies.

While it is well known that the GC-content of a probe can impact hybridization http://www.affymetrix.com/support/technical/technotes/customseq_arraybase_technote.pdf, [11] C-rich probes perform better than G-rich probes for the identical site when complementary strand quartets are compared [19] and fluorescence intensity declines with G-richness of a probe [20]. Additionally, bases within the G-stretch of a probe produce lower peak intensities, especially for stretches with ≥ 4 continuous Gs. It has previously been suggested that probes with multiple Gs in a row (G-stacks) tend to have higher cross-hybridization signals possibly caused by formation of G-quartets due to multiplex binding [21].

Taken together, the above findings imply that sites interrogated with G-rich probes may show stronger signal on the complementary strand employing C-rich probes. By applying additional computer algorithms (sPROFILER) to reduce GDAS/GSEQ no-calls by utilizing a distinct signal on either strand, 86% and 82% of the no-calls could be resolved for Harvard and Cincinnati arrays respectively while maintaining overall accuracy at ≥99.8%.

Mutations in GC-rich regions have previously been reported to be missed [13]. We also observed that variants that could be correctly identified were associated with lower GC-content probes as compared to those that were missed (Additional file 1). Adjusting probe length and positioning a known variable base at either end of the probe have been suggested to improve variant detection in GC-rich regions [13,30,27].

Small indels constitute nearly 24% of disease-causing mutations in the Human Gene Mutation Database as of November 2009 and have been shown to cause severe phenotypic variability [31-33]. The inability of resequencing arrays to have high sensitivity for detecting novel indels, especially those involving only a few base pairs, presents a significant limitation [10-12]. Regions showing aberrant hybridization patterns can be selected for confirmatory dideoxy sequencing to potentially detect variations (including indels) that are missed with resequencing arrays. However, as is evident from our data, small indels present challenges as they sometimes do not lead to easily discernible variability in hybridization patterns. After interrogating three different deletions on our arrays, we could detect only the largest deletion through a series of no-calls and a variant call in the region. Further algorithmic and technical improvements could entail development of a scheme for detection of indels by virtue of identifying a regional drop in signal intensity. Although we only observed a single intensity drop for one of the indels included in our validation, modification of the technical protocol to limit the secondary amplification of signal, originally employed to increase detection of low level transcripts in expression arrays, may not be necessary for resequencing arrays interrogating germline nuclear DNA variations present with at least 50% signal compared to wildtype. This protocol modification could lead to better discrimination of signal intensity drops across regions with an indel. In the interim, we have employed clinical oligo hybridization based sequencing technology only for diseases in which most mutations are substitutions (e.g. Noonan syndrome, cardiomyopathy) or the disease is recessive (e.g. hearing loss) in which detection is aimed at finding at least one of the pathogenic variants followed by capillary sequencing of the relevant gene to detect a second mutation that may have been missed.

Although a minor loss in analytical sensitivity is incurred through the use of hybridization based sequencing, this can be balanced with the increased efficiency and diminished cost of this technology compared to traditional approaches. Resequencing array-based mutation detection has been reported to produce a throughput of nearly 100 patients per technician per month and can thus be used as a method for initial genetic screening while being supplemented with conventional dideoxy sequencing for samples in which the array cannot identify a causative mutation [13]. In our hands, this technology has allowed us to cut the cost of testing roughly in half compared to dideoxy capillary sequencing approaches also employed in our clinical laboratories. As such, we have now implemented this technology in four different clinical tests including the HCM CardioChip, DCM CardioChip, Noonan Spectrum Chip, and OtoChip as described on http://pcpgm.partners.org/lmm.

## Conclusions

In conclusion, the described hearing loss gene chips represent the first resequencing arrays for molecular testing of nonsyndromic pediatric SNHL. Using the experimental protocols and additional computation algorithms described here, this technology provides a rapid, cost-effective and reasonably accurate method for identifying known and novel sequence variants in targeted DNA regions. However, follow-up sequencing required to resolve no-calls and false positives does limit the cost-effectiveness of the technology.

## Methods

### Patient enrolment

All 74 patient samples and clinical information were collected under IRB-approved protocols. Most cases (66) were recruited from clinical centers: 25 patients from Ohio (J. Greinwald), 20 from Massachusetts (H. Rehm and M. Kenna), 12 from Belgium (G. Van Camp), 5 from Israel (K. Avraham), 2 from Iowa (R. Smith), 2 from Nebraska (P. Kelley), and the remaining 8 represented anonymized samples obtained through clinical testing at the Laboratory for Molecular Medicine at the Partners Center for Personalized Genetic Medicine. All patients had previously tested negative for biallelic *GJB2 *mutations, except for 3 patients who had heterozygous mutations in the *GJB2 *gene. All patients had bilateral SNHL and were either recessive cases (e.g. with affected siblings) or singletons. DNA and clinical information was collected on most patients (de-identified clinical information for those cases that were anonymized) and included demographic information, audiometric profiles, family history, neuro-otologic history and physical examinations.

### Gene selection and array design

To determine what genes to place on a finite resequencing platform that was initially capable of accommodating less than 27,000 bp, we undertook a comprehensive literature search to estimate the likely frequency of mutations in genes known to cause childhood SNHL. Based on the prevalence of mutations in published populations and family studies, the inheritance pattern(s) of mutations (i.e. preference for recessive childhood hearing loss) and the impact of the gene on patient outcome (e.g. blindness developed due to Usher Syndrome), an initial set of genes were selected for inclusion on one or both hearing loss arrays (e.g. *GJB2, GJB6, CDH23, KCNE1, KCNQ1, MYO7A, OTOF, USH1C*, and *TMPRSS3*). Due to space limitations only the most conserved portion of *CDH23 *was tiled. Most other genes had extremely limited data on relative contribution and therefore several genes were selected based upon other minor characteristics (e.g. contribution to both dominant and recessive hearing loss (e.g. *MYO6*), interest in discovering a larger undocumented role in hearing loss (e.g. *SLC26A5*) and/or small gene size to fill remaining capacity on array (e.g. *TMIE*). It should be noted that current studies suggest that *SLC26A5 *mutations may not be a cause of hearing loss [34]. Details of the sequences included are in the **Methods **and Additional file 1.

Two arrays were designed, one at University of Cincinnati/Children's Hospital Medical Center (Cincinnati, OH) and one at Harvard Medical School (Boston, MA) with 8 genes on each array representing a total of 13 unique genes assessed. The Cincinnati array contained: *GJB2, GJB6, CDH23 *(59 out of 69 exons; 80.3% of the coding sequence), *KCNE1, KCNQ1, MYO7A, OTOF*, and *SLC26A4 *genes and the Harvard array contained: *GJB2, MYO6, MYO7A, OTOF, SLC26A5, TMIE, TMPRSS3*, and *USH1C *genes. For all genes, both the coding sequence and splice junctions were assessed. For the Cincinnati array, 2 bp of each flanking splice site were tiled for most exons. For the Harvard array, 10 bp of each flanking splice site were tiled for all exons. Additional file 1 contains an overview of the genes tiled on the arrays. Each array contained probes to interrogate roughly 26,000 bases of DNA (8 probes representing all 4 bases to assess the forward strand and all 4 bases to assess the reverse strand). In addition, probes designed to detect 17 previously reported insertions and deletions were also tiled on the Harvard array (Additional file 1).

### Array protocol

Initially the arrays were run according to the manufacturer's protocol (Affymetrix, Santa Clara, CA). Briefly, long range PCR conditions for the LA TaKaRa Polymerase (Takara, Japan) were: TaKaRa LA Taq 0.05 U/ul, 1× LA PCR Buffer II, 400 uM (each) dNTPs, 0.3 uM (each) primers, 4 ng/ul genomic DNA in a 25 ul reaction volume. Short range PCR conditions are described below for dideoxy sequencing. Cycling conditions for most reactions were 94°C 2 min, (94°C 15 sec, 68°C 9 min) × 30, 68°C 14 min, 4°C. Modifications using standard approaches to PCR optimization were made for some difficult reactions. All PCR assays were either quantified using PicoGreen (Molecular Probes, Eugene, OR) or absorbance spectrometry and then pooled in equimolar amounts. The PCR products were then purified, fragmented, labeled and hybridized to the array. Finally, the arrays were washed and scanned and the data were analyzed using the GeneChip DNA Analysis Software (GDAS 2.0, Affymetrix) or the GeneChip Sequence Analysis Software (GSEQ 4.0, Affymetrix). After initial experience, the Cincinnati protocol was modified to include only short range PCR in order to increase PCR and fragmentation consistency and facilitate the use of automation. In addition, PCR products were cleaned robotically using magnetic beads, 7.5× less fragmentation reagent was used than suggested (0.02 U vs the recommended 0.15 U) and fragmentation products were analyzed using an Agilent Bioanalyzer (Santa Clara, CA).

### Dideoxy DNA sequencing protocol

Complete dideoxy sequencing of all exons was performed for 14 of the Harvard patient arrays and all of the 25 Cincinnati arrays (enabling assessment of false negative rates) and partial sequencing was performed for the remaining 35 patients in order to confirm or clarify bases assigned as variants or "no calls" by Affymetrix GDAS or GSEQ software. PCR conditions: AmpliTaq Gold (Applied Biosystems, Foster City, CA) 0.05 U/ul, 1× ABI PCR Buffer, 2.5 mM MgCl2, 400 uM (each) dNTPs, 0.4 uM (each) primers, 2 ng/ul genomic DNA in a 25 ul reaction volume. Modifications using standards approaches to PCR optimization have been made for some difficult reactions. Cycling conditions are 94°C 2 min, (94°C 15 sec, 60°C 30 sec, 72°C 30 sec) × 30, 72°C 10 min, 4°C. Sequencing reactions are performed using the same primers for short-range PCR (or internal primers for long-range) in the following conditions: 0.25 μl Big Dye 3.1 (Applied Biosystems), 3.75 μl 5× Big Dye Buffer, 0.50 μl DMSO, 2 μl 10 μM primer, 2 ul template (1-5 ng) in 10 μl total volume. Cycle sequencing conditions are 94°C 4 min, (98°C 30 sec, 50°C 5 sec, 60°C 4 min) × 30, 4°C. Reactions are cleaned up using CleanSEQ magnetic beads (Agencourt Biosciences Corp, Beverly, MA) and then run on an ABI 3730 DNA Analyzer (Applied Biosystems). Chromatograms are analyzed using an automated Phred analysis program to check for quality followed by analysis using Sequencher 4.0 (GeneCodes, Ann Arbor, MI) and/or Mutation Surveyor (SoftGenetics, State College, PA) software.

### Data analysis

Affymetrix GDAS 2.0 with optimized settings (Additional file 1) was used for assignment of base calls for Harvard arrays and GSEQ 4.0 was used for Cincinnati arrays. The algorithm settings were determined after evaluating different quality score thresholds and comparing array calls with dideoxy sequencing results to characterize coverage and accuracy. The settings chosen were optimized for the highest call rate without increasing the false negative rate. We also developed a novel algorithm (sPROFILER) for strand-specific probe cell intensity comparison for filtering GDAS/GSEQ base calls. The algorithm was developed to re-examine GDAS/GSEQ output to reduce no-calls and, in some cases, false positives. sPROFILER attempts to resolve no-calls based on intensity signature from a single strand when a base cannot be called due to poor hybridization on one of the strands. The algorithm uses all wild-type bases within the array to determine threshold for peak to next highest intensity ratio on either strand and uses the base call at the position of interest across all arrays to determine the proportion of wild-type calls that are made on that position by GSEQ. The latter is used for scaling the threshold ratio and thus, in effect, making the algorithm more conservative while attempting to assign a wild-type call to a position that is being called variant in a large number of samples and vice-versa. We also adopted two additional bioinformatics filters developed for the *Francisella tularensis *whole-genome resequencing platform [17]. The filters were designed for reduction in false positives by screening variant calls that 1) are in regions rich in variant calls and no-calls, and 2) have low quality scores for the corresponding base call. With the exception of wild-type calls within no-call stretches, sPROFILER does not attempt to re-examine any base calls conforming to the reference sequence because GDAS and GSEQ are conservative in making wild-type calls and thus achieve low false negative rates. The output calls were compared against sequencing results and against GDAS/GSEQ calls for validation. sPROFILER was implemented in MATLAB. A detailed description of sPROFILER and accompanying MATLAB code are provided in Additional files 1 &2.

## Abbreviations

SNHL: sensorineural hearing loss; SNP: single nucleotide polymorphism; PCR: polymerase chain reaction; GDAS: GeneChip DNA Analysis Software; GSEQ: GeneChip Sequence Analysis Software; Indel: insertion/deletion; sPROFILER: strand-specific PRObe cell intensity comparison for FILtERing GDAS/GSEQ calls

## Authors' contributions

JE and AH performed DNA preparation and chip hybridization for Cincinnati arrays. AH analyzed patient-specific resequencing data and assisted in obtaining dideoxy sequencing reads. SC performed all laboratory work and raw data analysis for the Harvard arrays. MAK submitted patients for analysis on the Harvard arrays. HLR oversaw all work on the Harvard arrays. PK, JHG, BJA and HLR analyzed the resequencing data, designed the study, drafted and edited the manuscript. All authors read and approved the final manuscript.

## Supplementary Material

Additional file 1 **Supplementary data**. Provides accession numbers for genomic sequences tiled on the arrays, GDAS/GSEQ algorithm settings used for the analysis, a list of variants identified in hearing loss probands, and a detailed description of the sPROFILER algorithm. Click here for file

Additional file 2 **sPROFILER code**. Provides MATLAB code for sPROFILER, a novel algorithm implemented for improving array call rates. Provides a list of genes tiled on the arrays, variants identified in hearing loss probands, and a detailed description of the sPROFILER algorithm. Click here for file
