# Supplementary material for: High-throughput detection of mutations responsible for childhood hearing loss using resequencing microarrays
Source: BMC Biotechnol. 2010 Feb 10;10:10. doi: 10.1186/1472-6750-10-10 (PMC2841091; doi:10.1186/1472-6750-10-10)
Supplement: Additional file 1 — Supplementary data. Provides accession numbers for genomic sequences tiled on the arrays, GDAS/GSEQ algorithm settings used for the analysis, a list of variants identified in hearing loss probands, and a detailed description of the sPROFILER algorithm. [file 1472-6750-10-10-S1.PDF]

Supplementary Table 1. Genes selected for VDA analysis.

Gene	Locus	Accession Number	Length (bp)	Exons	Array
GJB2	DFNB1/A3	NM_004004	681	2	C H
OTOF	DFNB9	NM_194248	7051	48	C H
GJB6	DFNB1	NM_006783	783	1	C
SLC26A4	DFNB4/PDS	NM_000441	2342	20	C
KCNQ1	JLNS1	NM_000218	1746	14	C
KCNE1	JLNS2	NM_000219	390	1	C
SLC26A5	DFNB61	NM_198999	2057	18	H
TMPRSS3	DFNB8/10	NM_024022	1313	12	H
TMIE	DFNB6	NM_147196	468	4	H
MYO6	DFNB37/A22	NM_004999	3857	35	H
MYO7A	DFNB2/A11/USH1B	NM_000260	6647	49	C H
USH1C	DFNB18/USH1C	NM_153676	2730	28	H
CDH23	DFNB12/USH1D	NM_022124	10065	59	C*

C, Cincinnati array; **H**, Harvard array;
* 59 out of 69 exons were sequenced on the array, covering 80.3% of the CDH23 coding sequence. Exons 1, 58, 59, 60, 61, 62, 64, 65, 66 and 69 have been excluded.

Supplementary Table 2. Indel probes tiled on Harvard array for detection of indels previously reported in literature.

GJB2	35delG*
GJB2	167delT*
GJB2	235delC
GJB2	313_326del14
GJB2	334_335delAA
GJB2	631_632delTG
MYO6	36_37insT
MYO7A	1400_01insGCA
MYO7A	1595delA
MYO7A	2657_2665del9
MYO7A	3596_3597insT
OTOF	1651delG
TMIE	IVS1_2del6insC
TMIE	125_126insCGCC
TMPRSS3	207delC
USH1C	IVS5_2delA
USH1C	233_234insC

*At least one subject was found to carry the deletion.

Supplementary Table 3. GDAS 2.0/GSEQ 4.0 Settings

No Signal Threshold (probe signal/noise ratio)	1
Weak Signal Fold Threshold (mean/probe ratio)	20
Large SNR Threshold (probe signal/noise ratio)	20
Strand Quality Threshold (quality score)	-4
Total Quality Threshold (quality score)	30
Maximum Fraction of Heterozygote Calls (0-1)	0.9

Model Type (0=Heterozygote, 1=Homozygote) = 0	0
Perfect Call Quality Threshold (quality score)	2
Min Fraction of Calls in Neighboring Probes (0-1)	0.5
Min Fraction of Calls of Samples (0-1)	0.75

Supplementary Table 4. List of true variants identified in hearing loss probands.

Cincinnati Study

**Non-synonymous Changes**					
**Gene**	**CDS**	**AA**	**dbSNP ID**	**SNHL_Freq**	**Control_Freq**
CDH23	7C>T	R3C	rs7902757	5/74	8/100
CDH23	1098G>A	A366T		1/74	0/208
CDH23	1423G>A	V475M		2/74	2/208
CDH23	1469G>C	G490A	rs1227049	6/74	
CDH23	1487G>A	S496N	rs10999947	9/74	33/100
CDH23	1487G>A	S496N	rs10999947	9/74	33/100
CDH23	1621G>A	E541K		1/74	0/100
CDH23	3625A>G	T1209A	rs41281314	1/74	2/100
CDH23	4045C>T	R1349C	rs41281318	2/74	0/100
CDH23	4051A>G	N1351D	rs1227065	47/74	
CDH23	4310G>A	R1437Q		3/74	3/100
CDH23	4723G>A	A1575T	rs1227051	42/74	
CDH23	5023G>A	V1675I	rs17712523	11/74	
CDH23	5411G>A	R1804Q	rs3802711	8/74	19/100
CDH23	5418C>G	D1806E		2/74	3/100
CDH23	5650G>A	A1884T		1/74	0/100
CDH23	5996C>G	T1999S	rs11592462	29/74	
CDH23	6130G>A	E2044K	rs10466026	16/74	
CDH23	6275C>T	T2092I		1/74	0/100
CDH23	6847G>A	V2283I	rs41281334	4/74	
CDH23	7073G>A	F2358Q	rs4747194	16/74	
CDH23	7073G>A	R2358Q	rs4747194	16/74	
CDH23	7139C>T	P2380L	rs4747195	17/74	28/100
CDH23	7468G>A	E2490K	rs41281336	1/74	0/208
CDH23	7762G>C	E2588Q	rs41281338	1/74	2/100
CDH23	9728C>T	S3243F		1/74	0/100
CDH23	6402_6405del	E2135fs		2/74	0/100
GJB2	79G>A	V27I	rs2274084	2/74	
GJB2	101T>C	M34T	rs35887622	3/74	
GJB2	557C>T	T186M		1/74	
KCNE1	112A>G	S38G	rs17846179	33/74	
KCNE1	210G>T	K70N		1/74	
KCNE1	253G>A	D85N	rs1805128	1/74	2/100
MYO7A	47T>C	L16S	rs1052030	34/74	
MYO7A	268C>T	R90W		1/74	0/100
MYO7A	1028C>T	A343V		1/74	0/100
MYO7A	1232T>C	V411A		1/74	0/100

MYO7A	2617C>T	R873W		1/74	0/100
MYO7A	4589C>T	S1530L		1/74	0/100
MYO7A	4996A>T	S1666C	rs2276288	31/74	
MYO7A	5086C>T	R1696W		1/74	0/100
MYO7A	5730T>A	D1910E		1/74	0/100
MYO7A	6424G>A	D2142N	rs1132036	1/74	0/100
OTOF	158C>T	A53V	rs1879761	1/74	1/208
OTOF	244C>T	R82C	rs13031859	17/74	80/100
OTOF	244C>T	R82C	rs13031859	17/74	80/100
OTOF	2317C>T	R773C		2/74	1/208
OTOF	2702C>T	S901L		1/74	0/100
OTOF	3385G>A	V1129M		1/74	0/208
OTOF	3470G>A	R1157Q	rs56054534	1/74	0/100
OTOF	3608A>G	N1203S		3/74	2/208
OTOF	3917A>C	K1306T		2/74	0/208
OTOF	4936C>T	P1646S	rs17005371	6/74	3/100
OTOF	2401GA>TT	E801L		1/74	3/208
SLC26A4	535G>A	A179T		1/74	0/100
SLC26A4	777G>T	E259D		1/74	0/100
SLC26A4	898A>C	I300L		1/74	1/100
SLC26A4	1790T>C	L597S	rs55638457	1/74	1/100
SLC26A4	1826T>G	V609G	rs17154335	4/74	1/100
SLC26A4	2186T>C	L729P		1/74	0/100

**Synonymous and Intronic Changes**				
**Gene**	**CDS**	**AA**	**dbSNP ID**	**SNHL_Freq**
CDH23	366T>C	V122V	rs3802720	41/74
CDH23	1038G>A	P346P		1/74
CDH23	1053C>T	S351S	rs7903475	1/74
CDH23	2316T>C	N772N	rs3752752	36/74
CDH23	2388T>C	D796D	rs3752751	36/74
CDH23	2761C>T	L921L		1/74
CDH23	3009T>C	S1003S	rs10823829	4/74
CDH23	4287C>T	P1429P		1/74
CDH23	5100C>T	Y1700Y	rs10762480	10/74
CDH23	7572G>A	A2524A	rs10823849	17/74
CDH23	7630T>C	L2544L		1/74
CDH23	9873G>A	T3291T	rs2290021	6/74
KCNQ1	177C>T	A59A		2/74
KCNQ1	1185C>T	F395F		1/74
KCNQ1	1638G>A	S546S		11/74
MYO7A	288G>A	T96T	rs56023295	2/74
MYO7A	783T>C	G261G	rs762667	28/74
MYO7A	1605C>T	N535N		1/74
MYO7A	3246G>T	T1082T	rs35963362	1/74
MYO7A	3828G>A	S1276S		1/74
MYO7A	4755C>T	S1585S	rs7927472	28/74
MYO7A	4831C>T	L1611L		1/74

MYO7A	4845C>A	P1615P		1/74
MYO7A	4950C>T	N1650N		2/74
MYO7A	5598C>A	L1866L		1/74
MYO7A	5715A>G	K1905K	rs2276293	31/74
MYO7A	6240C>T	S2080S		3/74
MYO7A	6318G>A	K2106K	rs11237123	12/74
OTOF	372A>G	T124T	rs11687696	9/74
OTOF	387C>T	D129D		1/74
OTOF	945G>A	K315K	rs41288779	1/74
OTOF	1977G>A	P659P		1/74
OTOF	2022C>T	D674D	rs13004993	1/74
OTOF	2580C>G	V860V	rs2272069	14/74
OTOF	2613C>T	L871L	rs2272068	1/74
OTOF	2703G>A	S901S	rs4997760	1/74
OTOF	2736G>C	L912L	rs4335905	23/74
OTOF	2829C>T	G943G		1/74
OTOF	2829C>T	G943G		2/74
OTOF	4677G>A	V1559V	rs2272071	3/74
OTOF	5097C>T	I1699I	rs12386239	1/74

**Proband**	**Gene**	**CDS**	**AA**	**dbSNP ID**	**SNHL_Freq**	**Control**	**Het vs Hom**
1	CDH23	1487G>A	S496N	rs10999947	9/74	33/100	het
1	CDH23	2316T>C	N772N	rs3752752	36/74		het
1	CDH23	2388T>C	D796D	rs3752751	36/74		het
1	CDH23	4051A>G	N1351D	rs1227065	47/74		hom
1	CDH23	4723G>A	A1575T	rs1227051	42/74		hom
1	KCNE1	112A>G	S38G	rs17846179	33/74		hom
1	KCNQ1	1638G>A	S546S		11/74		het
1	MYO7A	47T>C	L16S	rs1052030	34/74		hom
1	OTOF	372A>G	T124T	rs11687696	9/74		het
2	CDH23	366T>C	V122V	rs3802720	41/74		het
2	CDH23	4051A>G	N1351D	rs1227065	47/74		hom
2	CDH23	4723G>A	A1575T	rs1227051	42/74		hom
2	KCNE1	112A>G	S38G	rs17846179	33/74		het
2	KCNE1	210G>T	K70N		1/74		het
2	MYO7A	47T>C	L16S	rs1052030	34/74		het
2	MYO7A	783T>C	G261G	rs762667	28/74		hom
2	OTOF	244C>T	R82C	rs13031859	17/74	80/100	het
4	CDH23	366T>C	V122V	rs3802720	41/74		hom
4	CDH23	1038G>A	P346P		1/74		het
4	CDH23	1487G>A	S496N	rs10999947	9/74	33/100	het
4	CDH23	2316T>C	N772N	rs3752752	36/74		hom
4	CDH23	2388T>C	D796D	rs3752751	36/74		hom
4	CDH23	4051A>G	N1351D	rs1227065	47/74		hom
4	CDH23	5996C>G	T1999S	rs11592462	29/74		hom
4	KCNE1	112A>G	S38G	rs17846179	33/74		het
4	MYO7A	47T>C	L16S	rs1052030	34/74		hom
4	MYO7A	783T>C	G261G	rs762667	28/74		het
4	MYO7A	4755C>T	S1585S	rs7927472	28/74		het
4	MYO7A	4996A>T	S1666C	rs2276288	31/74		het

4	MYO7A	5715A>G	K1905K	rs2276293	31/74		hom
6	CDH23	2316T>C	N772N	rs3752752	36/74		het
6	CDH23	2388T>C	D796D	rs3752751	36/74		het
6	CDH23	4051A>G	N1351D	rs1227065	47/74		het
6	CDH23	4723G>A	A1575T	rs1227051	42/74		het
6	CDH23	5418C>G	D1806E		2/74	3/100	het
6	CDH23	5996C>G	T1999S	rs11592462	29/74		het
6	KCNE1	112A>G	S38G	rs17846179	33/74		hom
6	MYO7A	783T>C	G261G	rs762667	28/74		het
6	MYO7A	4755C>T	S1585S	rs7927472	28/74		het
6	MYO7A	4996A>T	S1666C	rs2276288	31/74		het
6	MYO7A	5715A>G	K1905K	rs2276293	31/74		het
6	MYO7A	6240C>T	S2080S		3/74		het
6	MYO7A	6318G>A	K2106K	rs11237123	12/74		het
6	OTOF	2736G>C	L912L	rs4335905	23/74		het
7	CDH23	366T>C	V122V	rs3802720	41/74		het
7	CDH23	4051A>G	N1351D	rs1227065	47/74		het
7	CDH23	4723G>A	A1575T	rs1227051	42/74		het
7	CDH23	5023G>A	V1675I	rs17712523	11/74		het
7	CDH23	5100C>T	Y1700Y	rs10762480	10/74		het
7	CDH23	6130G>A	E2044K	rs10466026	16/74		het
7	CDH23	7139C>T	P2380L	rs4747195	17/74	28/100	het
7	CDH23	7468G>A	E2490K	rs41281336	1/74	0/208	het
7	CDH23	7572G>A	A2524A	rs10823849	17/74		het
7	MYO7A	783T>C	G261G	rs762667	28/74		het
7	MYO7A	4755C>T	S1585S	rs7927472	28/74		het
7	MYO7A	4996A>T	S1666C	rs2276288	31/74		het
7	OTOF	244C>T	R82C	rs13031859	17/74	80/100	hom
10	KCNE1	112A>G	S38G	rs17846179	33/74		het
10	KCNE1	253G>A	D85N	rs1805128	1/74	2/100	het
10	MYO7A	47T>C	L16S	rs1052030	34/74		het
10	MYO7A	4755C>T	S1585S	rs7927472	28/74		hom
10	MYO7A	4996A>T	S1666C	rs2276288	31/74		het
10	MYO7A	5715A>G	K1905K	rs2276293	31/74		hom
11	CDH23	366T>C	V122V	rs3802720	41/74		hom
11	CDH23	5100C>T	Y1700Y	rs10762480	10/74		het
11	CDH23	6130G>A	E2044K	rs10466026	16/74		het
11	CDH23	7073G>A	F2358Q	rs4747194	16/74		het
11	CDH23	7139C>T	P2380L	rs4747195	17/74	28/100	het
11	CDH23	7572G>A	A2524A	rs10823849	17/74		het
11	KCNE1	112A>G	S38G	rs17846179	33/74		hom
11	MYO7A	47T>C	L16S	rs1052030	34/74		het
11	MYO7A	783T>C	G261G	rs762667	28/74		het
11	MYO7A	4755C>T	S1585S	rs7927472	28/74		het
11	MYO7A	5715A>G	K1905K	rs2276293	31/74		het
11	OTOF	158C>T	A53V	rs1879761	1/74	1/208	het
13	CDH23	2316T>C	N772N	rs3752752	36/74		hom
13	CDH23	2388T>C	D796D	rs3752751	36/74		hom
13	CDH23	4051A>G	N1351D	rs1227065	47/74		hom
13	CDH23	4723G>A	A1575T	rs1227051	42/74		hom
13	CDH23	5100C>T	Y1700Y	rs10762480	10/74		het
13	CDH23	5411G>A	R1804Q	rs3802711	8/74	19/100	het
13	CDH23	6130G>A	E2044K	rs10466026	16/74		hom

13	CDH23	6847G>A	V2283I	rs41281334	4/74		het
13	CDH23	7073G>A	F2358Q	rs4747194	16/74		hom
13	CDH23	7139C>T	P2380L	rs4747195	17/74	28/100	hom
13	CDH23	7572G>A	A2524A	rs10823849	17/74		hom
13	MYO7A	47T>C	L16S	rs1052030	34/74		het
13	MYO7A	783T>C	G261G	rs762667	28/74		het
13	MYO7A	4755C>T	S1585S	rs7927472	28/74		het
13	MYO7A	4996A>T	S1666C	rs2276288	31/74		het
13	OTOF	244C>T	R82C	rs13031859	17/74	80/100	hom
13	SLC26A4	2186T>C	L729P		1/74	0/100	het
15	CDH23	366T>C	V122V	rs3802720	41/74		hom
15	CDH23	2316T>C	N772N	rs3752752	36/74		het
15	CDH23	2388T>C	D796D	rs3752751	36/74		het
15	CDH23	4051A>G	N1351D	rs1227065	47/74		het
15	CDH23	4723G>A	A1575T	rs1227051	42/74		het
15	CDH23	5996C>G	T1999S	rs11592462	29/74		hom
15	CDH23	7762G>C	E2588Q	rs41281338	1/74	2/100	het
15	KCNE1	112A>G	S38G	rs17846179	33/74		het
15	MYO7A	47T>C	L16S	rs1052030	34/74		het
15	MYO7A	783T>C	G261G	rs762667	28/74		het
15	MYO7A	4755C>T	S1585S	rs7927472	28/74		het
15	MYO7A	4996A>T	S1666C	rs2276288	31/74		het
15	MYO7A	5715A>G	K1905K	rs2276293	31/74		het
15	OTOF	372A>G	T124T	rs11687696	9/74		het
17	CDH23	366T>C	V122V	rs3802720	41/74		het
17	CDH23	1469G>C	G490A	rs1227049	6/74		het
17	CDH23	2316T>C	N772N	rs3752752	36/74		het
17	CDH23	2388T>C	D796D	rs3752751	36/74		het
17	CDH23	4051A>G	N1351D	rs1227065	47/74		hom
17	CDH23	4723G>A	A1575T	rs1227051	42/74		hom
17	CDH23	5023G>A	V1675I	rs17712523	11/74		het
17	CDH23	5100C>T	Y1700Y	rs10762480	10/74		het
17	CDH23	5411G>A	R1804Q	rs3802711	8/74	19/100	het
17	CDH23	5996C>G	T1999S	rs11592462	29/74		het
17	CDH23	6130G>A	E2044K	rs10466026	16/74		het
17	CDH23	7073G>A	F2358Q	rs4747194	16/74		het
17	CDH23	7139C>T	P2380L	rs4747195	17/74	28/100	het
17	CDH23	7572G>A	A2524A	rs10823849	17/74		het
17	MYO7A	47T>C	L16S	rs1052030	34/74		het
17	MYO7A	4755C>T	S1585S	rs7927472	28/74		hom
17	MYO7A	4996A>T	S1666C	rs2276288	31/74		hom
17	MYO7A	5715A>G	K1905K	rs2276293	31/74		hom
17	OTOF	244C>T	R82C	rs13031859	17/74	80/100	het
17	OTOF	372A>G	T124T	rs11687696	9/74		het
17	OTOF	2022C>T	D674D	rs13004993	1/74		het
17	OTOF	2580C>G	V860V	rs2272069	14/74		het
17	OTOF	2736G>C	L912L	rs4335905	23/74		het
21	CDH23	7C>T	R3C	rs7902757	5/74	8/100	het
21	CDH23	366T>C	V122V	rs3802720	41/74		het
21	CDH23	1423G>A	V475M		2/74	2/208	het
21	CDH23	1487G>A	S496N	rs10999947	9/74	33/100	het
21	CDH23	2316T>C	N772N	rs3752752	36/74		hom
21	CDH23	2388T>C	D796D	rs3752751	36/74		hom

21	CDH23	3009T>C	S1003S	rs10823829	4/74		het
21	CDH23	4045C>T	R1349C	rs41281318	2/74	0/100	het
21	CDH23	4051A>G	N1351D	rs1227065	47/74		hom
21	CDH23	4723G>A	A1575T	rs1227051	42/74		hom
21	CDH23	6130G>A	E2044K	rs10466026	16/74		het
21	CDH23	6847G>A	V2283I	rs41281334	4/74		het
21	CDH23	7073G>A	F2358Q	rs4747194	16/74		het
21	CDH23	7139C>T	P2380L	rs4747195	17/74	28/100	het
21	CDH23	7572G>A	A2524A	rs10823849	17/74		het
21	CDH23	9873G>A	T3291T	rs2290021	6/74		het
21	KCNE1	112A>G	S38G	rs17846179	33/74		hom
21	KCNQ1	1638G>A	S546S		11/74		het
21	MYO7A	47T>C	L16S	rs1052030	34/74		het
21	MYO7A	4755C>T	S1585S	rs7927472	28/74		het
21	MYO7A	4996A>T	S1666C	rs2276288	31/74		het
21	MYO7A	5715A>G	K1905K	rs2276293	31/74		het
21	MYO7A	6318G>A	K2106K	rs11237123	12/74		het
21	OTOF	3385G>A	V1129M		1/74	0/208	het
21	OTOF	3608A>G	N1203S		3/74	2/208	het
21	OTOF	3917A>C	K1306T		2/74	0/208	hom
21	OTOF	4677G>A	V1559V	rs2272071	3/74		het
21	SLC26A4	1826T>G	V609G	rs17154335	4/74	1/100	het
23	CDH23	2316T>C	N772N	rs3752752	36/74		hom
23	CDH23	2388T>C	D796D	rs3752751	36/74		hom
23	CDH23	4051A>G	N1351D	rs1227065	47/74		hom
23	CDH23	4723G>A	A1575T	rs1227051	42/74		hom
23	CDH23	5996C>G	T1999S	rs11592462	29/74		het
23	KCNQ1	1185C>T	F395F		1/74		het
23	MYO7A	47T>C	L16S	rs1052030	34/74		het
23	MYO7A	783T>C	G261G	rs762667	28/74		hom
23	MYO7A	4755C>T	S1585S	rs7927472	28/74		het
23	MYO7A	4950C>T	N1650N		2/74		het
23	MYO7A	4996A>T	S1666C	rs2276288	31/74		het
23	MYO7A	5715A>G	K1905K	rs2276293	31/74		het
23	MYO7A	6318G>A	K2106K	rs11237123	12/74		het
23	OTOF	244C>T	R82C	rs13031859	17/74	80/100	het
23	OTOF	2580C>G	V860V	rs2272069	14/74		het
23	OTOF	2736G>C	L912L	rs4335905	23/74		het
25	CDH23	2316T>C	N772N	rs3752752	36/74		het
25	CDH23	2388T>C	D796D	rs3752751	36/74		het
25	CDH23	5100C>T	Y1700Y	rs10762480	10/74		het
25	CDH23	5411G>A	R1804Q	rs3802711	8/74	19/100	het
25	CDH23	5996C>G	T1999S	rs11592462	29/74		het
25	CDH23	6130G>A	E2044K	rs10466026	16/74		het
25	CDH23	7073G>A	F2358Q	rs4747194	16/74		het
25	CDH23	7139C>T	P2380L	rs4747195	17/74	28/100	het
25	CDH23	7572G>A	A2524A	rs10823849	17/74		het
25	KCNE1	112A>G	S38G	rs17846179	33/74		het
25	MYO7A	47T>C	L16S	rs1052030	34/74		hom
25	MYO7A	4996A>T	S1666C	rs2276288	31/74		hom
25	MYO7A	5715A>G	K1905K	rs2276293	31/74		hom
26	CDH23	366T>C	V122V	rs3802720	41/74		hom
26	CDH23	1487G>A	S496N	rs10999947	9/74		het

26	CDH23	4051A>G	N1351D	rs1227065	47/74		hom
26	CDH23	4310G>A	R1437Q		3/74	3/100	het
26	CDH23	4723G>A	A1575T	rs1227051	42/74		hom
26	KCNQ1	1638G>A	S546S		11/74		het
26	OTOF	244C>T	R82C	rs13031859	17/74		het
26	OTOF	372A>G	T124T	rs11687696	9/74		het
30	MYO7A	47T>C	L16S	rs1052030	34/74		hom
30	MYO7A	4755C>T	S1585S	rs7927472	28/74		het
30	MYO7A	4996A>T	S1666C	rs2276288	31/74		het
30	MYO7A	5086C>T	R1696W		1/74	0/100	het
30	MYO7A	5715A>G	K1905K	rs2276293	31/74		het
30	MYO7A	6318G>A	K2106K	rs11237123	12/74		het
30	SLC26A4	1790T>C	L597S	rs55638457	1/74	1/100	het
32	CDH23	366T>C	V122V	rs3802720	41/74		het
32	CDH23	2316T>C	N772N	rs3752752	36/74		het
32	CDH23	2388T>C	D796D	rs3752751	36/74		het
32	CDH23	4051A>G	N1351D	rs1227065	47/74		hom
32	CDH23	4723G>A	A1575T	rs1227051	42/74		hom
32	CDH23	5100C>T	Y1700Y	rs10762480	10/74		hom
32	CDH23	5411G>A	R1804Q	rs3802711	8/74	19/100	hom
32	CDH23	6130G>A	E2044K	rs10466026	16/74		hom
32	CDH23	7073G>A	F2358Q	rs4747194	16/74		hom
32	CDH23	7139C>T	P2380L	rs4747195	17/74	28/100	hom
32	CDH23	7572G>A	A2524A	rs10823849	17/74		hom
32	KCNE1	112A>G	S38G	rs17846179	33/74		het
32	MYO7A	47T>C	L16S	rs1052030	34/74		het
32	OTOF	244C>T	R82C	rs13031859	17/74	80/100	hom
32	OTOF	2702C>T	S901L		1/74	0/100	het
35	CDH23	366T>C	V122V	rs3802720	41/74		hom
35	CDH23	1469G>C	G490A	rs1227049	6/74		het
35	CDH23	1487G>A	S496N	rs10999947	9/74	33/100	het
35	CDH23	2316T>C	N772N	rs3752752	36/74		hom
35	CDH23	2388T>C	D796D	rs3752751	36/74		hom
35	CDH23	4051A>G	N1351D	rs1227065	47/74		hom
35	CDH23	4723G>A	A1575T	rs1227051	42/74		hom
35	CDH23	5023G>A	V1675I	rs17712523	11/74		het
35	CDH23	5100C>T	Y1700Y	rs10762480	10/74		het
35	CDH23	5411G>A	R1804Q	rs3802711	8/74	19/100	het
35	CDH23	5996C>G	T1999S	rs11592462	29/74		het
35	CDH23	6130G>A	E2044K	rs10466026	16/74		het
35	CDH23	7073G>A	F2358Q	rs4747194	16/74		het
35	CDH23	7139C>T	P2380L	rs4747195	17/74	28/100	het
35	CDH23	7572G>A	A2524A	rs10823849	17/74		het
35	CDH23	7630T>C	L2544L		1/74		het
35	CDH23	9728C>T	S3243F		1/74	0/100	het
35	GJB2	101T>C	M34T	rs35887622	3/74		het
35	KCNE1	112A>G	S38G	rs17846179	33/74		het
35	KCNQ1	1638G>A	S546S		11/74		het
35	MYO7A	47T>C	L16S	rs1052030	34/74		het
35	MYO7A	4755C>T	S1585S	rs7927472	28/74		het
35	MYO7A	4845C>A	P1615P		1/74		het
35	MYO7A	4996A>T	S1666C	rs2276288	31/74		het
35	MYO7A	5715A>G	K1905K	rs2276293	31/74		het

35	OTOF	387C>T	D129D		1/74		het
35	OTOF	2580C>G	V860V	rs2272069	14/74		het
35	OTOF	2736G>C	L912L	rs4335905	23/74		het
37	CDH23	366T>C	V122V	rs3802720	41/74		het
37	CDH23	1053C>T	S351S	rs7903475	1/74		het
37	CDH23	2316T>C	N772N	rs3752752	36/74		het
37	CDH23	2388T>C	D796D	rs3752751	36/74		het
37	CDH23	5418C>G	D1806E		2/74	3/100	het
37	CDH23	5996C>G	T1999S	rs11592462	29/74		het
37	CDH23	6275C>T	T2092I		1/74	0/100	het
37	MYO7A	47T>C	L16S	rs1052030	34/74		het
37	MYO7A	783T>C	G261G	rs762667	28/74		het
37	MYO7A	4755C>T	S1585S	rs7927472	28/74		hom
37	MYO7A	4996A>T	S1666C	rs2276288	31/74		hom
37	MYO7A	5715A>G	K1905K	rs2276293	31/74		hom
37	OTOF	2580C>G	V860V	rs2272069	14/74		het
37	OTOF	2736G>C	L912L	rs4335905	23/74		het
37	OTOF	4936C>T	P1646S	rs17005371	6/74	3/100	het
37	OTOF	2401GA>TT	E801L		1/74	3/208	het
38	CDH23	2316T>C	N772N	rs3752752	36/74		hom
38	CDH23	2388T>C	D796D	rs3752751	36/74		hom
38	CDH23	4051A>G	N1351D	rs1227065	47/74		hom
38	CDH23	4723G>A	A1575T	rs1227051	42/74		hom
38	CDH23	5023G>A	V1675I	rs17712523	11/74		hom
38	CDH23	5996C>G	T1999S	rs11592462	29/74		hom
38	CDH23	6402_6405del	E2135fs		2/74	0/100	hom
38	GJB2	79G>A	V27I	rs2274084	2/74		hom
38	KCNE1	112A>G	S38G	rs17846179	33/74		hom
38	KCNQ1	1638G>A	S546S		11/74		het
38	MYO7A	47T>C	L16S	rs1052030	34/74		het
38	MYO7A	4996A>T	S1666C	rs2276288	31/74		het
38	MYO7A	5715A>G	K1905K	rs2276293	31/74		het
41	CDH23	366T>C	V122V	rs3802720	41/74		hom
41	CDH23	5996C>G	T1999S	rs11592462	29/74		hom
41	KCNE1	112A>G	S38G	rs17846179	33/74		hom
41	MYO7A	783T>C	G261G	rs762667	28/74		hom
42	CDH23	7C>T	R3C	rs7902757	5/74	8/100	het
42	CDH23	366T>C	V122V	rs3802720	41/74		hom
42	CDH23	1423G>A	V475M		2/74	2/208	het
42	CDH23	2316T>C	N772N	rs3752752	36/74		het
42	CDH23	2388T>C	D796D	rs3752751	36/74		het
42	CDH23	3009T>C	S1003S	rs10823829	4/74		het
42	CDH23	4051A>G	N1351D	rs1227065	47/74		hom
42	CDH23	4723G>A	A1575T	rs1227051	42/74		hom
42	CDH23	5996C>G	T1999S	rs11592462	29/74		het
42	CDH23	6130G>A	E2044K	rs10466026	16/74		het
42	CDH23	7073G>A	R2358Q	rs4747194	16/74		het
42	CDH23	7139C>T	P2380L	rs4747195	17/74	28/100	het
42	CDH23	7572G>A	A2524A	rs10823849	17/74		het
42	CDH23	9873G>A	T3291T	rs2290021	6/74		het
42	KCNE1	112A>G	S38G	rs17846179	33/74		het
42	MYO7A	47T>C	L16S	rs1052030	34/74		hom
42	MYO7A	783T>C	G261G	rs762667	28/74		hom

42	MYO7A	3828G>A	S1276S		1/74		het
42	MYO7A	4589C>T	S1530L		1/74	0/100	het
42	MYO7A	4755C>T	S1585S	rs7927472	28/74		het
42	MYO7A	4996A>T	S1666C	rs2276288	31/74		hom
42	MYO7A	5715A>G	K1905K	rs2276293	31/74		hom
42	MYO7A	6318G>A	K2106K	rs11237123	12/74		het
42	OTOF	2613C>T	L871L	rs2272068	1/74		het
42	OTOF	2703G>A	S901S	rs4997760	1/74		het
42	OTOF	2736G>C	L912L	rs4335905	23/74		hom
42	OTOF	2829C>T	G943G		1/74		het
42	OTOF	3608A>G	N1203S		3/74	2/208	het
42	OTOF	4677G>A	V1559V	rs2272071	3/74		het
42	OTOF	4936C>T	P1646S	rs17005371	6/74	3/100	het
44	KCNE1	112A>G	S38G	rs17846179	33/74		het
44	MYO7A	47T>C	L16S	rs1052030	34/74		het
44	MYO7A	288G>A	T96T	rs56023295	2/74		het
44	MYO7A	5730T>A	D1910E		1/74	0/100	het
44	OTOF	2736G>C	L912L	rs4335905	23/74		hom
45	CDH23	1487G>A	S496N	rs10999947	9/74	33/100	het
45	CDH23	2316T>C	N772N	rs3752752	36/74		het
45	CDH23	2388T>C	D796D	rs3752751	36/74		het
45	CDH23	4051A>G	N1351D	rs1227065	47/74		het
45	CDH23	4723G>A	A1575T	rs1227051	42/74		het
45	CDH23	5996C>G	T1999S	rs11592462	29/74		het
45	CDH23	6130G>A	E2044K	rs10466026	16/74		het
45	CDH23	6847G>A	V2283I	rs41281334	4/74		het
45	CDH23	7073G>A	F2358Q	rs4747194	16/74		het
45	CDH23	7139C>T	P2380L	rs4747195	17/74	28/100	het
45	CDH23	7572G>A	A2524A	rs10823849	17/74		het
45	CDH23	9873G>A	T3291T	rs2290021	6/74		het
45	KCNE1	112A>G	S38G	rs17846179	33/74		hom
45	KCNQ1	1638G>A	S546S		11/74		het
45	MYO7A	47T>C	L16S	rs1052030	34/74		het
45	MYO7A	783T>C	G261G	rs762667	28/74		het
45	MYO7A	4755C>T	S1585S	rs7927472	28/74		het
45	MYO7A	4996A>T	S1666C	rs2276288	31/74		het
45	MYO7A	5715A>G	K1905K	rs2276293	31/74		het
45	MYO7A	6318G>A	K2106K	rs11237123	12/74		het
46	CDH23	7C>T	R3C	rs7902757	5/74	8/100	het
46	CDH23	366T>C	V122V	rs3802720	41/74		hom
46	CDH23	1469G>C	G490A	rs1227049	6/74		het
46	CDH23	2316T>C	N772N	rs3752752	36/74		hom
46	CDH23	2388T>C	D796D	rs3752751	36/74		hom
46	CDH23	3009T>C	S1003S	rs10823829	4/74		het
46	CDH23	4051A>G	N1351D	rs1227065	47/74		het
46	CDH23	4310G>A	R1437Q		3/74	3/100	het
46	CDH23	4723G>A	A1575T	rs1227051	42/74		het
46	CDH23	5023G>A	V1675I	rs17712523	11/74		het
46	CDH23	5996C>G	T1999S	rs11592462	29/74		het
46	MYO7A	47T>C	L16S	rs1052030	34/74		het
46	MYO7A	268C>T	R90W		1/74	0/100	het
46	MYO7A	5598C>A	L1866L		1/74		het
46	OTOF	244C>T	R82C	rs13031859	17/74	80/100	het

46	OTOF	1977G>A	P659P		1/74		het
46	OTOF	2580C>G	V860V	rs2272069	14/74		het
46	OTOF	2736G>C	L912L	rs4335905	23/74		het
46	OTOF	3470G>A	R1157Q	rs56054534	1/74	0/100	het
48	CDH23	366T>C	V122V	rs3802720	41/74		hom
48	CDH23	1098G>A	A366T		1/74	0/208	het
48	CDH23	4051A>G	N1351D	rs1227065	47/74		het
48	CDH23	4723G>A	A1575T	rs1227051	42/74		het
48	MYO7A	47T>C	L16S	rs1052030	34/74		het
48	MYO7A	6240C>T	S2080S		3/74		het
48	MYO7A	6318G>A	K2106K	rs11237123	12/74		het
48	OTOF	244C>T	R82C	rs13031859	17/74		het
48	SLC26A4	777G>T	E259D		1/74	0/100	het
49	CDH23	366T>C	V122V	rs3802720	41/74		het
49	CDH23	1469G>C	G490A	rs1227049	6/74		het
49	CDH23	2316T>C	N772N	rs3752752	36/74		het
49	CDH23	2388T>C	D796D	rs3752751	36/74		het
49	CDH23	4051A>G	N1351D	rs1227065	47/74		hom
49	CDH23	4723G>A	A1575T	rs1227051	42/74		hom
49	CDH23	5996C>G	T1999S	rs11592462	29/74		het
49	CDH23	6130G>A	E2044K	rs10466026	16/74		het
49	CDH23	6847G>A	V2283I	rs41281334	4/74		het
49	CDH23	7073G>A	F2358Q	rs4747194	16/74		het
49	CDH23	7139C>T	P2380L	rs4747195	17/74	28/100	het
49	CDH23	7572G>A	A2524A	rs10823849	17/74		het
49	CDH23	9873G>A	T3291T	rs2290021	6/74		het
49	KCNE1	112A>G	S38G	rs17846179	33/74		het
49	MYO7A	783T>C	G261G	rs762667	28/74		het
49	MYO7A	6318G>A	K2106K	rs11237123	12/74		het
50	KCNE1	112A>G	S38G	rs17846179	33/74		hom
50	KCNQ1	1638G>A	S546S		11/74		het
50	MYO7A	47T>C	L16S	rs1052030	34/74		het
50	MYO7A	783T>C	G261G	rs762667	28/74		het
50	MYO7A	1605C>T	N535N		1/74		het
50	MYO7A	4755C>T	S1585S	rs7927472	28/74		hom
50	MYO7A	4996A>T	S1666C	rs2276288	31/74		hom
50	MYO7A	5715A>G	K1905K	rs2276293	31/74		hom
51	CDH23	7C>T	R3C	rs7902757	5/74	8/100	hom
51	CDH23	366T>C	V122V	rs3802720	41/74		hom
51	CDH23	2316T>C	N772N	rs3752752	36/74		hom
51	CDH23	2388T>C	D796D	rs3752751	36/74		hom
51	CDH23	3009T>C	S1003S	rs10823829	4/74		het
51	CDH23	3625A>G	T1209A	rs41281314	1/74	2/100	het
51	CDH23	4051A>G	N1351D	rs1227065	47/74		hom
51	CDH23	4310G>A	R1437Q		3/74	3/100	het
51	CDH23	7073G>A	F2358Q	rs4747194	16/74		het
51	CDH23	7139C>T	P2380L	rs4747195	17/74	28/100	het
51	CDH23	7572G>A	A2524A	rs10823849	17/74		het
51	CDH23	9873G>A	T3291T	rs2290021	6/74		het
51	GJB2	101T>C	M34T	rs35887622	3/74		het
51	KCNQ1	177C>T	A59A		2/74		hom
51	MYO7A	47T>C	L16S	rs1052030	34/74		hom
51	MYO7A	783T>C	G261G	rs762667	28/74		hom

51	MYO7A	1028C>T	A343V		1/74	0/100	het
51	MYO7A	4755C>T	S1585S	rs7927472	28/74		het
51	MYO7A	4831C>T	L1611L		1/74		het
51	MYO7A	4950C>T	N1650N		2/74		het
51	MYO7A	4996A>T	S1666C	rs2276288	31/74		het
51	MYO7A	5715A>G	K1905K	rs2276293	31/74		het
51	MYO7A	6318G>A	K2106K	rs11237123	12/74		het
51	MYO7A	6424G>A	D2142N	rs1132036	1/74	0/100	het
51	OTOF	945G>A	K315K	rs41288779	1/74		het
51	OTOF	2736G>C	L912L	rs4335905	23/74		hom
51	OTOF	2829C>T	G943G		2/74		hom
51	OTOF	4936C>T	P1646S	rs17005371	6/74	3/100	hom
51	SLC26A4	1826T>G	V609G	rs17154335	4/74	1/100	het
53	CDH23	366T>C	V122V	rs3802720	41/74		hom
53	CDH23	4051A>G	N1351D	rs1227065	47/74		het
53	CDH23	4723G>A	A1575T	rs1227051	42/74		het
53	CDH23	5996C>G	T1999S	rs11592462	29/74		het
53	KCNQ1	1638G>A	S546S		11/74		het
53	MYO7A	47T>C	L16S	rs1052030	34/74		het
53	MYO7A	4755C>T	S1585S	rs7927472	28/74		het
53	MYO7A	4996A>T	S1666C	rs2276288	31/74		het
53	MYO7A	5715A>G	K1905K	rs2276293	31/74		het
53	OTOF	372A>G	T124T	rs11687696	9/74		hom
53	OTOF	2580C>G	V860V	rs2272069	14/74		hom
53	OTOF	2736G>C	L912L	rs4335905	23/74		hom
54	CDH23	366T>C	V122V	rs3802720	41/74		hom
54	CDH23	1469G>C	G490A	rs1227049	6/74		het
54	CDH23	2316T>C	N772N	rs3752752	36/74		het
54	CDH23	2388T>C	D796D	rs3752751	36/74		het
54	CDH23	4051A>G	N1351D	rs1227065	47/74		het
54	CDH23	4723G>A	A1575T	rs1227051	42/74		het
54	CDH23	5023G>A	V1675I	rs17712523	11/74		het
54	CDH23	5996C>G	T1999S	rs11592462	29/74		het
54	KCNE1	112A>G	S38G	rs17846179	33/74		het
54	MYO7A	2617C>T	R873W		1/74	0/100	het
54	MYO7A	4755C>T	S1585S	rs7927472	28/74		het
54	MYO7A	4996A>T	S1666C	rs2276288	31/74		het
54	OTOF	3608A>G	N1203S		3/74	2/208	het
54	OTOF	4677G>A	V1559V	rs2272071	3/74		het
54	OTOF	4936C>T	P1646S	rs17005371	6/74	3/100	het
54	OTOF	5097C>T	I1699I	rs12386239	1/74		het
54	SLC26A4	898A>C	I300L		1/74	1/100	het
54	SLC26A4	1826T>G	V609G	rs17154335	4/74	1/100	het
55	CDH23	2316T>C	N772N	rs3752752	36/74		hom
55	CDH23	2388T>C	D796D	rs3752751	36/74		hom
55	CDH23	4051A>G	N1351D	rs1227065	47/74		hom
55	CDH23	4723G>A	A1575T	rs1227051	42/74		hom
55	CDH23	5100C>T	Y1700Y	rs10762480	10/74		het
55	CDH23	5411G>A	R1804Q	rs3802711	8/74	19/100	het
55	CDH23	5996C>G	T1999S	rs11592462	29/74		het
55	CDH23	6130G>A	E2044K	rs10466026	16/74		het
55	CDH23	7073G>A	F2358Q	rs4747194	16/74		het
55	CDH23	7139C>T	P2380L	rs4747195	17/74	28/100	het

55	CDH23	7572G>A	A2524A	rs10823849	17/74		het
55	MYO7A	47T>C	L16S	rs1052030	34/74		het
55	MYO7A	783T>C	G261G	rs762667	28/74		het
55	MYO7A	3246G>T	T1082T	rs35963362	1/74		het
55	MYO7A	4755C>T	S1585S	rs7927472	28/74		het
55	MYO7A	4996A>T	S1666C	rs2276288	31/74		het
55	MYO7A	5715A>G	K1905K	rs2276293	31/74		het
55	MYO7A	6318G>A	K2106K	rs11237123	12/74		het
55	OTOF	244C>T	R82C	rs13031859	17/74	80/100	het
55	OTOF	372A>G	T124T	rs11687696	9/74		het
55	OTOF	2580C>G	V860V	rs2272069	14/74		het
55	OTOF	2736G>C	L912L	rs4335905	23/74		het
56	CDH23	366T>C	V122V	rs3802720	41/74		hom
56	CDH23	1621G>A	E541K		1/74	0/100	het
56	CDH23	4051A>G	N1351D	rs1227065	47/74		het
56	CDH23	4723G>A	A1575T	rs1227051	42/74		het
56	CDH23	5023G>A	V1675I	rs17712523	11/74		het
56	CDH23	5996C>G	T1999S	rs11592462	29/74		het
56	GJB2	557C>T	T186M		1/74		het
56	KCNQ1	1638G>A	S546S		11/74		het
56	MYO7A	783T>C	G261G	rs762667	28/74		hom
56	MYO7A	1232T>C	V411A		1/74	0/100	het
56	MYO7A	4755C>T	S1585S	rs7927472	28/74		het
56	MYO7A	4996A>T	S1666C	rs2276288	31/74		het
56	MYO7A	5715A>G	K1905K	rs2276293	31/74		het
56	MYO7A	6240C>T	S2080S		3/74		het
56	MYO7A	6318G>A	K2106K	rs11237123	12/74		het
56	OTOF	372A>G	T124T	rs11687696	9/74		het
56	OTOF	2580C>G	V860V	rs2272069	14/74		hom
56	OTOF	2736G>C	L912L	rs4335905	23/74		hom
58	CDH23	366T>C	V122V	rs3802720	41/74		het
58	CDH23	1469G>C	G490A	rs1227049	6/74		het
58	CDH23	2316T>C	N772N	rs3752752	36/74		het
58	CDH23	2388T>C	D796D	rs3752751	36/74		het
58	CDH23	4051A>G	N1351D	rs1227065	47/74		het
58	CDH23	4723G>A	A1575T	rs1227051	42/74		het
58	CDH23	5023G>A	V1675I	rs17712523	11/74		hom
58	CDH23	5650G>A	A1884T		1/74	0/100	het
58	CDH23	5996C>G	T1999S	rs11592462	29/74		hom
58	GJB2	101T>C	M34T	rs35887622	3/74		het
58	KCNQ1	1638G>A	S546S		11/74		het
58	MYO7A	47T>C	L16S	rs1052030	34/74		hom
58	OTOF	244C>T	R82C	rs13031859	17/74	80/100	het
58	OTOF	2580C>G	V860V	rs2272069	14/74		hom
58	OTOF	2736G>C	L912L	rs4335905	23/74		hom
59	CDH23	366T>C	V122V	rs3802720	41/74		hom
59	CDH23	2316T>C	N772N	rs3752752	36/74		hom
59	CDH23	2388T>C	D796D	rs3752751	36/74		hom
59	CDH23	2761C>T	L921L		1/74		het
59	CDH23	4051A>G	N1351D	rs1227065	47/74		hom
59	CDH23	4723G>A	A1575T	rs1227051	42/74		het
59	CDH23	5023G>A	V1675I	rs17712523	11/74		het
59	CDH23	5996C>G	T1999S	rs11592462	29/74		hom

59	KCNE1	112A>G	S38G	rs17846179	33/74		hom
59	MYO7A	783T>C	G261G	rs762667	28/74		het
59	MYO7A	4755C>T	S1585S	rs7927472	28/74		het
59	MYO7A	4996A>T	S1666C	rs2276288	31/74		het
59	MYO7A	5715A>G	K1905K	rs2276293	31/74		het
59	MYO7A	6318G>A	K2106K	rs11237123	12/74		het
61	CDH23	366T>C	V122V	rs3802720	41/74		hom
61	CDH23	1487G>A	S496N	rs10999947	9/74	33/100	het
61	CDH23	2316T>C	N772N	rs3752752	36/74		het
61	CDH23	2388T>C	D796D	rs3752751	36/74		het
61	CDH23	4051A>G	N1351D	rs1227065	47/74		het
61	CDH23	4287C>T	P1429P		1/74		het
61	CDH23	4723G>A	A1575T	rs1227051	42/74		het
61	CDH23	5100C>T	Y1700Y	rs10762480	10/74		het
61	CDH23	5411G>A	R1804Q	rs3802711	8/74	19/100	het
61	CDH23	5996C>G	T1999S	rs11592462	29/74		het
61	CDH23	6130G>A	E2044K	rs10466026	16/74		het
61	CDH23	7073G>A	F2358Q	rs4747194	16/74		het
61	CDH23	7139C>T	P2380L	rs4747195	17/74	28/100	het
61	CDH23	7572G>A	A2524A	rs10823849	17/74		het
61	MYO7A	783T>C	G261G	rs762667	28/74		hom
61	MYO7A	4996A>T	S1666C	rs2276288	31/74		het
61	MYO7A	5715A>G	K1905K	rs2276293	31/74		het
61	OTOF	244C>T	R82C	rs13031859	17/74	80/100	het
61	OTOF	2580C>G	V860V	rs2272069	14/74		hom
61	OTOF	2736G>C	L912L	rs4335905	23/74		hom
61	SLC26A4	535G>A	A179T		1/74	0/100	het
61	SLC26A4	1826T>G	V609G	rs17154335	4/74	1/100	het
153	CDH23	366T>C	V122V	rs3802720	41/74		hom
153	CDH23	1487G>A	S496N	rs10999947	9/74	33/100	hom
153	CDH23	2316T>C	N772N	rs3752752	36/74		hom
153	CDH23	2388T>C	D796D	rs3752751	36/74		hom
153	CDH23	4045C>T	R1349C	rs41281318	2/74	0/100	het
153	CDH23	4051A>G	N1351D	rs1227065	47/74		hom
153	CDH23	4723G>A	A1575T	rs1227051	42/74		hom
153	CDH23	5996C>G	T1999S	rs11592462	29/74		het
153	CDH23	6130G>A	E2044K	rs10466026	16/74		het
153	CDH23	7073G>A	F2358Q	rs4747194	16/74		het
153	CDH23	7139C>T	P2380L	rs4747195	17/74	28/100	het
153	CDH23	7572G>A	A2524A	rs10823849	17/74		het
153	CDH23	9873G>A	T3291T	rs2290021	6/74		het
153	KCNE1	112A>G	S38G	rs17846179	33/74		hom
153	MYO7A	47T>C	L16S	rs1052030	34/74		het
153	MYO7A	783T>C	G261G	rs762667	28/74		hom
153	MYO7A	4755C>T	S1585S	rs7927472	28/74		het
153	MYO7A	4996A>T	S1666C	rs2276288	31/74		het
153	MYO7A	5715A>G	K1905K	rs2276293	31/74		het
153	OTOF	244C>T	R82C	rs13031859	17/74	80/100	het
153	OTOF	372A>G	T124T	rs11687696	9/74		het
153	OTOF	2317C>T	R773C		2/74	1/208	het
153	OTOF	2736G>C	L912L	rs4335905	23/74		het
153	OTOF	4936C>T	P1646S	rs17005371	6/74	3/100	het
241	CDH23	366T>C	V122V	rs3802720	41/74		het

241	CDH23	2316T>C	N772N	rs3752752	36/74		het
241	CDH23	2388T>C	D796D	rs3752751	36/74		het
241	CDH23	4051A>G	N1351D	rs1227065	47/74		hom
241	CDH23	4723G>A	A1575T	rs1227051	42/74		hom
241	CDH23	5996C>G	T1999S	rs11592462	29/74		het
241	KCNE1	112A>G	S38G	rs17846179	33/74		hom
241	KCNQ1	1638G>A	S546S		11/74		het
241	MYO7A	288G>A	T96T	rs56023295	2/74		het
241	MYO7A	4755C>T	S1585S	rs7927472	28/74		het
241	MYO7A	4996A>T	S1666C	rs2276288	31/74		het
241	MYO7A	5715A>G	K1905K	rs2276293	31/74		het
241	OTOF	244C>T	R82C	rs13031859	17/74	80/100	het
241	OTOF	2317C>T	R773C		2/74	1/208	het
241	OTOF	2736G>C	L912L	rs4335905	23/74		het

Harvard Study

Non-synonymous Changes

Gene	NT Change	AA Change	rsID	Freq	Control_freq
GJB2	35delG	G12fs		3/28	
GJB2	79G>A	V27I	rs2274084	1/28	
GJB2	167delT	L56fs		1/28	
GJB2	380G>A	R127H		1/28	
GJB2	457G>A	V153I		2/28	
MYO7A	47T>C	L16S	rs1052030	23/41	
MYO7A	380T>C	I127T	rs41298131	1/34	
MYO7A	970G>T	A324S		1/34	
MYO7A	2002C>T	R668C		1/34	
MYO7A	2500C>T	R834C		1/34	0/94
MYO7A	4127A>G	K1376R		1/34	
MYO7A	4697C>T	T1566M	rs41298747	1/34	
MYO7A	4996A>T	S1666C	rs2276288	31/41	
MYO7A	5156A>G	Y1719C		4/41	
MYO7A	5860C>A	L1954I	rs948962	33/41	
MYO7A	5866G>A	V1956I		1/28	
MYO7A	6640G>A	G2214S		1/34	
OTOF	244C>T	R82C	rs13031859	29/41	
OTOF	506G>A	R169Q		1/34	
OTOF	1194T>A	D398E		1/41	
OTOF	1630C>T	R544C		1/41	
OTOF	1723G>A	V575M	rs55676840	1/34	
OTOF	2317C>T	R773C		1/41	
OTOF	2348delG	G782fs		2/41	
OTOF	2464C>T	R822W		2/41	2/94
OTOF	2888G>A	R963Q		2/41	
OTOF	2908C>T	R970C		2/41	
OTOF	3247G>C	A1083P		2/41	
OTOF	3470G>A	R1157Q	rs56054534	1/34	
OTOF	3751T>G	C1251G	rs41288773	1/34	
OTOF	4582G>A	D1528N		1/34	

OTOF	4936C>T	P1646S	rs17005371	3/41	
OTOF	5332G>T	V1778F		1/41	
OTOF	5558G>A	R1853Q		1/34	
SLC26A5	137T>C	L46P		1/28	
SLC26A5	1610T>C	I537T		1/28	0/94
TMIE	367AAG[7_9]	K123[7_9]	rs34038267	19/29	
TMPRSS3	157G>A	V53I	rs928302	3/34	
TMPRSS3	268G>A	A90T	rs45598239	1/34	
TMPRSS3	280G>A	G94R		1/34	0/94
TMPRSS3	331G>A	G111S	rs35227181	9/41	
TMPRSS3	757A>G	I2531V	rs2839500	9/41	
TMPRSS3	1042G>A	D348N		1/34	
TMPRSS3	1180_1187delins(b-sat)			1/28	
TMPRSS3	1211C>T	P404L	rs28939084	1/28	
USH1C	2457C>G	D819E	rs1064074	30/41	
**Synonymous and Intronic Changes**					
MYO6	1176A>G	T392T	rs2273857	1/41	
MYO6	1722C>T	D574D	rs11756446	6/28	
MYO6	2946+8T>C			1/37	
MYO6	2982G>A	E994E	rs55905349	1/28	
MYO7A	78G>A	A26A		1/41	
MYO7A	288G>A	T96T	rs56023295	1/41	
MYO7A	468C>T	I156I	rs12420129	3/34	
MYO7A	486C>T	A162A		1/28	
MYO7A	783T>C	G261G	rs762667	29/41	
MYO7A	4023C>T	P1341P		1/28	
MYO7A	4074C>T	S1358S		1/34	
MYO7A	4461C>T	N1487N	rs56174006	1/34	
MYO7A	4755T>C	S1585S	rs7927472	31/41	
MYO7A	4983C>T	D1661D		1/34	
MYO7A	5598C>A	L1866L		1/28	
MYO7A	5619G>A	R1872R	rs45450893	4/34	
MYO7A	5715A>G	K1905K	rs2276293	27/41	
MYO7A	5857-7A>T		rs1320703	31/36	
MYO7A	6240C>T	S2080S		3/34	
MYO7A	6318G>A	K2106K	rs11237123	19/41	
MYO7A	6519C>T	N2173N		2/41	
OTOF	372A>G	T124T	rs11687696	24/41	
OTOF	945G>A	K315K	rs41288779	5/34	
OTOF	1926C>T	N642N		3/41	
OTOF	2022C>T	D674D	rs13004993	3/34	
OTOF	2025G>A	E675E		2/34	
OTOF	2580C>G	V860V	rs2272069	18/41	
OTOF	2613C>T	L871L	rs2272068	3/41	
OTOF	2703G>A	S901S	rs4997760	3/41	
OTOF	2736G>C	L912L	rs4335905	22/41	
OTOF	3189G>A	A1063A		2/41	

OTOF	4332C>T	T1444T		2/41
OTOF	4677G>A	V1559V	rs2272071	3/41
OTOF	5391C>T	F1797F		1/34
OTOF	5655C>T	R1885R	rs45442103	7/41
SLC26A5	1302A>G	S434S		1/41
SLC26A5	1335T>C	I445I		1/41
TMPRSS3	453G>A	V151V	rs2839501	29/41
TMPRSS3	617-1_617-2insTA		rs34966432	6/32
TMPRSS3	789C>T	Y263Y		1/41
USH1C	114C>T	D38D		1/34
USH1C	381G>T	G127G	rs41282942	2/34
USH1C	1188A>G	P396P	rs2240487	36/41
USH1C	1770C>T	A590A	rs17776775	4/34
USH1C	2340C>T	V780V	rs10832796	14/41

**Proband**	**Gene**	**CDS**	**AA**	**dbSNP ID**	**SNHL/Afreq**	**Control**	**Het vs. Hom**
1	MYO7A	783T>C	G261G	rs762667	29/41		het
1	MYO7A	4755C>T	S1585S	rs7927472	31/41		het
1	MYO7A	4996A>T	S1666C	rs2276288	31/41		het
1	MYO7A	5715A>G	K1905K	rs2276293	27/41		het
1	MYO7A	5857-7A>T		rs1320703	33/41		
1	MYO7A	5860C>A	L1954I	rs948962	33/41		het
1	OTOF	244C>T	R82C	rs13031859	29/41		hom
1	OTOF	372A>G	T124T	rs11687696	24/41		het
1	OTOF	1926C>T	N642N		3/41		het
1	OTOF	2464C>T	R822W		2/41	2/94	het
1	OTOF	2736G>C	L912L	rs4335905	22/41		het
1	OTOF	3189G>A	A1063A		2/41		het
1	OTOF	3247G>C	A1083P		2/41		het
1	OTOF	4332C>T	T1444T		2/41		het
1	TMIE	367AAG[7_9]	K123[7_9]	rs34038267	19/29		
1	TMPRSS3	331G>A	G111S	rs35227181	9/41		het
1	TMPRSS3	453G>A	V151V	rs2839501	29/41		het
1	USH1C	1188A>G	P396P	rs2240487	36/41		het
1	USH1C	2340C>T	V780V	rs10832796	14/41		het
1	USH1C	2457C>G	D819E	rs1064074	30/41		het
2	GJB2	35delG	G12fs		3/28		
2	MYO7A	783T>C	G261G	rs762667	29/41		hom
2	MYO7A	4023C>T	P1341P		1/28		het
2	MYO7A	4755C>T	S1585S	rs7927472	31/41		het
2	MYO7A	4996A>T	S1666C	rs2276288	31/41		hom
2	MYO7A	5156A>G	Y1719C		4/41		het
2	MYO7A	5715A>G	K1905K	rs2276293	27/41		het
2	MYO7A	5857-7A>T		rs1320703	33/41		
2	MYO7A	5860C>A	L1954I	rs948962	33/41		het
2	MYO7A	6318G>A	K2106K	rs11237123	19/41		het
2	OTOF	372A>G	T124T	rs11687696	24/41		het
2	OTOF	2580C>G	V860V	rs2272069	18/41		het
2	OTOF	2736G>C	L912L	rs4335905	22/41		het
2	OTOF	4582G>A	D1528N		1/34		het
2	TMIE	367AAG[7_9]	K123[7_9]	rs34038267	19/29		
2	TMPRSS3	453G>A	V151V	rs2839501	29/41		het

2	USH1C	1188A>G	P396P	rs2240487	36/41		het
2	USH1C	2457C>G	D819E	rs1064074	30/41		het
3	MYO7A	47T>C	L16S	rs1052030	23/41		hom
3	MYO7A	2500C>T	R834C		1/34	0/94	het
3	MYO7A	4755C>T	S1585S	rs7927472	31/41		het
3	MYO7A	4996A>T	S1666C	rs2276288	31/41		het
3	MYO7A	5715A>G	K1905K	rs2276293	27/41		het
3	MYO7A	5857-7A>T		rs1320703	33/41		
3	MYO7A	5860C>A	L1954I	rs948962	33/41		het
3	TMIE	367AAG[7_9]	K123[7_9]	rs34038267	19/29		het
3	USH1C	381G>T	G127G	rs41282942	2/34		het
3	USH1C	1188A>G	P396P	rs2240487	36/41		het
3	USH1C	2340C>T	V780V	rs10832796	14/41		hom
3	USH1C	2457C>G	D819E	rs1064074	30/41		het
3	OTOF	244C>T	R82C	rs13031859	29/41		hom
3	OTOF	372A>G	T124T	rs11687696	24/41		het
3	OTOF	2580C>G	V860V	rs2272069	18/41		het
3	OTOF	2613C>T	L871L	rs2272068	3/41		het
3	OTOF	2703G>A	S901S	rs4997760	3/41		het
3	OTOF	2736G>C	L912L	rs4335905	22/41		hom
3	OTOF	4677G>A	V1559V	rs2272071	3/41		het
4	MYO7A	47T>C	L16S	rs1052030	23/41		hom
4	MYO7A	783T>C	G261G	rs762667	29/41		het
4	MYO7A	4755C>T	S1585S	rs7927472	31/41		hom
4	MYO7A	4996A>T	S1666C	rs2276288	31/41		het
4	MYO7A	5156A>G	Y1719C		4/41		het
4	MYO7A	5715A>G	K1905K	rs2276293	27/41		het
4	MYO7A	5857-7A>T		rs1320703	33/41		
4	MYO7A	5860C>A	L1954I	rs948962	33/41		hom
4	TMPRSS3	453G>A	V151V	rs2839501	29/41		het
4	TMPRSS3	757A>G	I2531V	rs2839500	9/41		het
4	USH1C	1188A>G	P396P	rs2240487	36/41		hom
4	USH1C	2457C>G	D819E	rs1064074	30/41		het
4	OTOF	244C>T	R82C	rs13031859	29/41		het
4	OTOF	372A>G	T124T	rs11687696	24/41		het
4	OTOF	1926C>T	N642N		3/41		het
5	MYO7A	47T>C	L16S	rs1052030	23/41		het
5	MYO7A	783T>C	G261G	rs762667	29/41		het
5	MYO7A	4996A>T	S1666C	rs2276288	31/41		hom
5	MYO7A	5715A>G	K1905K	rs2276293	27/41		hom
5	MYO7A	5857-7A>T		rs1320703	33/41		
5	MYO7A	5860C>A	L1954I	rs948962	33/41		hom
5	MYO7A	6240C>T	S2080S		3/34		het
5	MYO7A	6318G>A	K2106K	rs11237123	19/41		het
5	TMPRSS3	1180_1187delins(b-sat)			1/28		hom
5	TMIE	367AAG[7_9]	K123[7_9]	rs34038267	19/29		het
5	USH1C	1188A>G	P396P	rs2240487	36/41		het
5	USH1C	1770C>T	A590A	rs17776775	4/34		het
5	USH1C	2457C>G	D819E	rs1064074	30/41		het
5	OTOF	244C>T	R82C	rs13031859	29/41		het
5	OTOF	372A>G	T124T	rs11687696	24/41		het
5	MYO6	1722C>T	D574D	rs11756446	6/28		het
6	MYO7A	783T>C	G261G	rs762667	29/41		het
6	MYO7A	4697C>T	T1566M	rs41298747	1/34		het
6	MYO7A	4755C>T	S1585S	rs7927472	31/41		het

6	MYO7A	4996A>T	S1666C	rs2276288	31/41		het
6	MYO7A	5715A>G	K1905K	rs2276293	27/41		het
6	MYO7A	5857-7A>T		rs1320703	33/41		
6	MYO7A	5860C>A	L1954I	rs948962	33/41		het
6	MYO7A	6318G>A	K2106K	rs11237123	19/41		het
6	TMPRSS3	453G>A	V151V	rs2839501	29/41		hom
6	TMIE	367AAG[7_9]	K123[7_9]	rs34038267	19/29		
6	USH1C	1188A>G	P396P	rs2240487	36/41		het
6	USH1C	1770C>T	A590A	rs17776775	4/34		hom
6	USH1C	2457C>G	D819E	rs1064074	30/41		hom
6	OTOF	244C>T	R82C	rs13031859	29/41		hom
6	OTOF	2025G>A	E675E		2/34		het
6	OTOF	2580C>G	V860V	rs2272069	18/41		hom
6	OTOF	2736G>C	L912L	rs4335905	22/41		hom
6	GJB2	457G>A	V153I		2/28		het
7	MYO7A	4755C>T	S1585S	rs7927472	31/41		hom
7	TMIE	367AAG[7_9]	K123[7_9]	rs34038267	19/29		
7	TMPRSS3	331G>A	G111S	rs35227181	9/41		het
7	TMPRSS3	453G>A	V151V	rs2839501	29/41		het
7	USH1C	1188A>G	P396P	rs2240487	36/41		hom
7	USH1C	2457C>G	D819E	rs1064074	30/41		hom
7	OTOF	372A>G	T124T	rs11687696	24/41		hom
7	OTOF	2580C>G	V860V	rs2272069	18/41		hom
7	OTOF	2736G>C	L912L	rs4335905	22/41		hom
8	MYO7A	783T>C	G261G	rs762667	29/41		het
8	MYO7A	4755C>T	S1585S	rs7927472	31/41		het
8	MYO7A	4996A>T	S1666C	rs2276288	31/41		het
8	MYO7A	5715A>G	K1905K	rs2276293	27/41		het
8	MYO7A	5857-7A>T		rs1320703	33/41		
8	MYO7A	5860C>A	L1954I	rs948962	33/41		het
8	TMIE	367AAG[7_9]	K123[7_9]	rs34038267	19/29		
8	TMPRSS3	453G>A	V151V	rs2839501	29/41		het
8	USH1C	2340C>T	V780V	rs10832796	14/41		het
8	OTOF	2580C>G	V860V	rs2272069	18/41		het
8	OTOF	2736G>C	L912L	rs4335905	22/41		hom
8	OTOF	4936C>T	P1646S	rs17005371	3/41		het
8	GJB2	35delG	G12fs		3/28		het
8	GJB2	167delT	L56fs		1/28		het
9	MYO7A	47T>C	L16S	rs1052030	23/41		het
9	MYO7A	783T>C	G261G	rs762667	29/41		het
9	MYO7A	4755C>T	S1585S	rs7927472	31/41		het
9	MYO7A	4996A>T	S1666C	rs2276288	31/41		het
9	MYO7A	5619G>A	R1872R	rs45450893	4/34		het
9	MYO7A	5715A>G	K1905K	rs2276293	27/41		het
9	MYO7A	5857-7A>T		rs1320703	33/41		
9	MYO7A	5860C>A	L1954I	rs948962	33/41		het
9	MYO7A	6318G>A	K2106K	rs11237123	19/41		het
9	TMIE	367AAG[7_9]	K123[7_9]	rs34038267	19/29		
9	TMPRSS3	331G>A	G111S	rs35227181	9/41		hom
9	USH1C	1188A>G	P396P	rs2240487	36/41		hom
9	USH1C	2457C>G	D819E	rs1064074	30/41		het
9	MYO6	1722C>T	D574D	rs11756446	6/28		het
9	OTOF	244C>T	R82C	rs13031859	29/41		hom
9	OTOF	2613C>T	L871L	rs2272068	3/41		het
9	OTOF	2703G>A	S901S	rs4997760	3/41		het

9	OTOF	2736G>C	L912L	rs4335905	22/41		het
9	OTOF	4677G>A	V1559V	rs2272071	3/41		het
9	GJB2	35delG	G12fs		3/28	hom	hom
10	MYO7A	783T>C	G261G	rs762667	29/41		hom
10	MYO7A	4755C>T	S1585S	rs7927472	31/41		het
10	MYO7A	4996A>T	S1666C	rs2276288	31/41		het
10	MYO7A	5857-7A>T		rs1320703	33/41		
10	MYO7A	5860C>A	L1954I	rs948962	33/41		het
10	MYO7A	6318G>A	K2106K	rs11237123	19/41		het
10	TMPRSS3	157G>A	V53I	rs928302	3/34		het
10	TMPRSS3	453G>A	V151V	rs2839501	29/41		het
10	USH1C	1188A>G	P396P	rs2240487	36/41		hom
10	USH1C	2457C>G	D819E	rs1064074	30/41		hom
10	MYO6	1722C>T	D574D	rs11756446	6/28		het
10	OTOF	372A>G	T124T	rs11687696	24/41		het
10	OTOF	2022C>T	D674D	rs13004993	3/34		het
10	OTOF	2580C>G	V860V	rs2272069	18/41		hom
10	OTOF	2736G>C	L912L	rs4335905	22/41		hom
11	MYO7A	4996A>T	S1666C	rs2276288	31/41		hom
11	MYO7A	5619G>A	R1872R	rs45450893	4/34		het
11	MYO7A	5857-7A>T		rs1320703	33/41		
11	MYO7A	5860C>A	L1954I	rs948962	33/41		hom
11	MYO7A	6318G>A	K2106K	rs11237123	19/41		het
11	TMPRSS3	453G>A	V151V	rs2839501	29/41		hom
11	USH1C	1188A>G	P396P	rs2240487	36/41		hom
11	USH1C	2340C>T	V780V	rs10832796	14/41		het
11	USH1C	2457C>G	D819E	rs1064074	30/41		hom
11	OTOF	2580C>G	V860V	rs2272069	18/41		het
11	OTOF	2736G>C	L912L	rs4335905	22/41		het
12	MYO7A	47T>C	L16S	rs1052030	23/41		het
12	MYO7A	783T>C	G261G	rs762667	29/41		het
12	MYO7A	4755C>T	S1585S	rs7927472	31/41		het
12	MYO7A	4996A>T	S1666C	rs2276288	31/41		het
12	MYO7A	5715A>G	K1905K	rs2276293	27/41		het
12	MYO7A	5857-7A>T		rs1320703	33/41		
12	MYO7A	5860C>A	L1954I	rs948962	33/41		het
12	TMIE	367AAG[7_9]	K123[7_9]	rs34038267	19/29		het
12	TMPRSS3	453G>A	V151V	rs2839501	29/41		het
12	USH1C	1188A>G	P396P	rs2240487	36/41		het
12	USH1C	2457C>G	D819E	rs1064074	30/41		het
12	OTOF	244C>T	R82C	rs13031859	29/41		het
12	OTOF	372A>G	T124T	rs11687696	24/41		het
12	OTOF	2022C>T	D674D	rs13004993	3/34		het
12	OTOF	2580C>G	V860V	rs2272069	18/41		het
12	OTOF	2736G>C	L912L	rs4335905	22/41		het
13	MYO7A	47T>C	L16S	rs1052030	23/41		het
13	MYO7A	4755C>T	S1585S	rs7927472	31/41		hom
13	SLC26A5	1610T>C	I537T		1/28	0/94	
13	TMIE	367AAG[7_9]	K123[7_9]	rs34038267	19/29		
13	TMPRSS3	331G>A	G111S	rs35227181	9/41		het
13	TMPRSS3	453G>A	V151V	rs2839501	29/41		het
13	USH1C	1188A>G	P396P	rs2240487	36/41		het
13	OTOF	244C>T	R82C	rs13031859	29/41		het
13	OTOF	945G>A	K315K	rs41288779	5/34		het
14	MYO7A	47T>C	L16S	rs1052030	23/41		het

14	MYO7A	783T>C	G261G	rs762667	29/41		het
14	MYO7A	4755C>T	S1585S	rs7927472	31/41		hom
14	TMIE	367AAG[7_9]	K123[7_9]	rs34038267	19/29		het
14	TMPRSS3	280G>A	G94R		1/34	0/94	het
14	TMPRSS3	453G>A	V151V	rs2839501	29/41		het
14	USH1C	2340C>T	V780V	rs10832796	14/41		het
14	MYO6	1722C>T	D574D	rs11756446	6/28		het
14	OTOF	244C>T	R82C	rs13031859	29/41		hom
15	MYO6	2982G>A	E994E	rs55905349	1/28		het
15	MYO7A	783T>C	G261G	rs762667	29/41		hom
15	MYO7A	4996A>T	S1666C	rs2276288	31/41		hom
15	MYO7A	5715A>G	K1905K	rs2276293	27/41		hom
15	MYO7A	5857-7A>T		rs1320703	33/41		
15	MYO7A	5860C>A	L1954I	rs948962	33/41		hom
15	OTOF	244C>T	R82C	rs13031859	29/41		het
15	OTOF	372A>G	T124T	rs11687696	24/41		het
15	OTOF	2580C>G	V860V	rs2272069	18/41		het
15	OTOF	2736G>C	L912L	rs4335905	22/41		het
15	OTOF	5391C>T	F1797F		1/34		het
15	TMPRSS3	453G>A	V151V	rs2839501	29/41		het
15	USH1C	1188A>G	P396P	rs2240487	36/41		het
16	MYO7A	468C>T	I156I	rs12420129	3/34		het
16	MYO7A	783T>C	G261G	rs762667	29/41		het
16	MYO7A	4996A>T	S1666C	rs2276288	31/41		hom
16	MYO7A	5715A>G	K1905K	rs2276293	27/41		hom
16	MYO7A	5857-7A>T		rs1320703	33/41		
16	MYO7A	5860C>A	L1954I	rs948962	33/41		het
16	MYO7A	5866G>A	V1956I		1/28		het
16	MYO7A	6318G>A	K2106K	rs11237123	19/41		het
16	OTOF	244C>T	R82C	rs13031859	29/41		hom
16	OTOF	5558G>A	R1853Q		1/34		het
16	OTOF	5655C>T	R1885R	rs45442103	7/41		het
16	TMIE	367AAG[7_9]	K123[7_9]	rs34038267	19/29		
16	USH1C	1188A>G	P396P	rs2240487	36/41		het
16	USH1C	1770C>T	A590A	rs17776775	4/34		het
16	USH1C	2457C>G	D819E	rs1064074	30/41		het
17	GJB2	457G>A	V153I		2/28		het
17	MYO6	1722C>T	D574D	rs11756446	6/28		het
17	MYO7A	783T>C	G261G	rs762667	29/41		hom
17	MYO7A	4755C>T	S1585S	rs7927472	31/41		het
17	MYO7A	4996A>T	S1666C	rs2276288	31/41		het
17	MYO7A	5619G>A	R1872R	rs45450893	4/34		het
17	MYO7A	5715A>G	K1905K	rs2276293	27/41		het
17	MYO7A	5857-7A>T		rs1320703	33/41		
17	MYO7A	5860C>A	L1954I	rs948962	33/41		het
17	MYO7A	6318G>A	K2106K	rs11237123	19/41		het
17	OTOF	244C>T	R82C	rs13031859	29/41		het
17	OTOF	372A>G	T124T	rs11687696	24/41		het
17	OTOF	945G>A	K315K	rs41288779	5/34		het
17	TMIE	367AAG[7_9]	K123[7_9]	rs34038267	19/29		het
17	TMPRSS3	617-1_617-2insTA		rs34966432	6/32		
17	USH1C	1188A>G	P396P	rs2240487	36/41		het
17	USH1C	2457C>G	D819E	rs1064074	30/41		het
18	GJB2	380G>A	R127H		1/28		het
18	MYO7A	468C>T	I156I	rs12420129	3/34		hom

18	MYO7A	783T>C	G261G	rs762667	29/41		hom
18	MYO7A	4996A>T	S1666C	rs2276288	31/41		hom
18	OTOF	244C>T	R82C	rs13031859	29/41		het
18	OTOF	2580C>G	V860V	rs2272069	18/41		hom
18	OTOF	2736G>C	L912L	rs4335905	22/41		hom
18	TMPRSS3	617-1_617-2insTA		rs34966432	6/32		
18	TMPRSS3	757A>G	I2531V	rs2839500	9/41		het
18	USH1C	1188A>G	P396P	rs2240487	36/41		het
18	USH1C	2340C>T	V780V	rs10832796	14/41		het
18	USH1C	2457C>G	D819E	rs1064074	30/41		het
19	GJB2	79G>A	V27I	rs2274084	1/28		het
19	MYO7A	47T>C	L16S	rs1052030	23/41		het
19	MYO7A	783T>C	G261G	rs762667	29/41		het
19	MYO7A	4996A>T	S1666C	rs2276288	31/41		hom
19	MYO7A	5715A>G	K1905K	rs2276293	27/41		hom
19	MYO7A	5857-7A>T		rs1320703	33/41		
19	MYO7A	5860C>A	L1954I	rs948962	33/41		het
19	MYO7A	6318G>A	K2106K	rs11237123	19/41		het
19	OTOF	244C>T	R82C	rs13031859	29/41		hom
19	OTOF	372A>G	T124T	rs11687696	24/41		het
19	OTOF	5655C>T	R1885R	rs45442103	7/41		het
19	TMIE	367AAG[7_9]	K123[7_9]	rs34038267	19/29		
19	USH1C	1188A>G	P396P	rs2240487	36/41		het
19	USH1C	2340C>T	V780V	rs10832796	14/41		het
19	USH1C	2457C>G	D819E	rs1064074	30/41		het
20	MYO7A	783T>C	G261G	rs762667	29/41		het
20	MYO7A	4755C>T	S1585S	rs7927472	31/41		hom
20	OTOF	244C>T	R82C	rs13031859	29/41		het
20	OTOF	945G>A	K315K	rs41288779	5/34		het
20	TMIE	367AAG[7_9]	K123[7_9]	rs34038267	19/29		
20	TMPRSS3	331G>A	G111S	rs35227181	9/41		het
20	USH1C	1188A>G	P396P	rs2240487	36/41		hom
21	MYO6	1722C>T	D574D	rs11756446	6/28		het
21	MYO7A	47T>C	L16S	rs1052030	23/41		het
21	MYO7A	486C>T	A162A		1/28		het
21	MYO7A	4755C>T	S1585S	rs7927472	31/41		hom
21	MYO7A	6240C>T	S2080S		3/34		het
21	MYO7A	6318G>A	K2106K	rs11237123	19/41		het
21	OTOF	244C>T	R82C	rs13031859	29/41		hom
21	OTOF	372A>G	T124T	rs11687696	24/41		het
21	OTOF	5655C>T	R1885R	rs45442103	7/41		het
21	TMPRSS3	453G>A	V151V	rs2839501	29/41		het
21	USH1C	1188A>G	P396P	rs2240487	36/41		hom
21	USH1C	2457C>G	D819E	rs1064074	30/41		hom
22	MYO7A	47T>C	L16S	rs1052030	23/41		hom
22	MYO7A	783T>C	G261G	rs762667	29/41		het
22	MYO7A	4755C>T	S1585S	rs7927472	31/41		hom
22	MYO7A	5857-7A>T		rs1320703	33/41		
22	MYO7A	5860C>A	L1954I	rs948962	33/41		het
22	MYO7A	6318G>A	K2106K	rs11237123	19/41		het
22	OTOF	372A>G	T124T	rs11687696	24/41		het
22	TMPRSS3	453G>A	V151V	rs2839501	29/41		hom
22	USH1C	1188A>G	P396P	rs2240487	36/41		hom
22	USH1C	2457C>G	D819E	rs1064074	30/41		hom
23	MYO7A	47T>C	L16S	rs1052030	23/41		het

23	MYO7A	4996A>T	S1666C	rs2276288	31/41		hom
23	MYO7A	5715A>G	K1905K	rs2276293	27/41		hom
23	MYO7A	5857-7A>T		rs1320703	33/41		
23	MYO7A	5860C>A	L1954I	rs948962	33/41		hom
23	OTOF	244C>T	R82C	rs13031859	29/41		hom
23	OTOF	372A>G	T124T	rs11687696	24/41		het
23	OTOF	1723G>A	V575M	rs55676840	1/34		het
23	OTOF	3470G>A	R1157Q	rs56054534	1/34		het
23	USH1C	1188A>G	P396P	rs2240487	36/41		het
23	USH1C	2340C>T	V780V	rs10832796	14/41		het
23	USH1C	2457C>G	D819E	rs1064074	30/41		het
24	MYO7A	47T>C	L16S	rs1052030	23/41		het
24	MYO7A	783T>C	G261G	rs762667	29/41		het
24	MYO7A	4755C>T	S1585S	rs7927472	31/41		hom
24	OTOF	244C>T	R82C	rs13031859	29/41		het
24	TMPRSS3	331G>A	G111S	rs35227181	9/41		het
24	TMPRSS3	453G>A	V151V	rs2839501	29/41		het
24	TMPRSS3	757A>G	I2531V	rs2839500	9/41		het
24	USH1C	1188A>G	P396P	rs2240487	36/41		het
24	USH1C	2340C>T	V780V	rs10832796	14/41		het
24	USH1C	2457C>G	D819E	rs1064074	30/41		het
25	MYO7A	47T>C	L16S	rs1052030	23/41		hom
25	MYO7A	4755C>T	S1585S	rs7927472	31/41		het
25	MYO7A	4996A>T	S1666C	rs2276288	31/41		het
25	MYO7A	5715A>G	K1905K	rs2276293	27/41		het
25	MYO7A	5857-7A>T		rs1320703	33/41		
25	MYO7A	5860C>A	L1954I	rs948962	33/41		het
25	MYO7A	6318G>A	K2106K	rs11237123	19/41		het
25	OTOF	372A>G	T124T	rs11687696	24/41		hom
25	OTOF	2580C>G	V860V	rs2272069	18/41		het
25	OTOF	2736G>C	L912L	rs4335905	22/41		het
25	TMPRSS3	453G>A	V151V	rs2839501	29/41		het
25	USH1C	1188A>G	P396P	rs2240487	36/41		hom
26	MYO7A	47T>C	L16S	rs1052030	23/41		het
26	MYO7A	4755C>T	S1585S	rs7927472	31/41		het
26	MYO7A	4996A>T	S1666C	rs2276288	31/41		het
26	MYO7A	5715A>G	K1905K	rs2276293	27/41		het
26	MYO7A	5857-7A>T		rs1320703	33/41		
26	MYO7A	5860C>A	L1954I	rs948962	33/41		het
26	MYO7A	6318G>A	K2106K	rs11237123	19/41		het
26	OTOF	244C>T	R82C	rs13031859	29/41		hom
26	OTOF	2736G>C	L912L	rs4335905	22/41		het
26	OTOF	4936C>T	P1646S	rs17005371	3/41		het
26	OTOF	5655C>T	R1885R	rs45442103	7/41		het
26	TMPRSS3	1211C>T	P404L	rs28939084	1/28		hom
26	TMPRSS3	1211C>T	P404L	rs28939084	1/28		hom
26	USH1C	1188A>G	P396P	rs2240487	36/41		het
26	USH1C	2457C>G	D819E	rs1064074	30/41		het

Supplementary Figure 1. Relationship between number of bases covered by a PCR fragment and the mean hybridization intensity of the fragment.

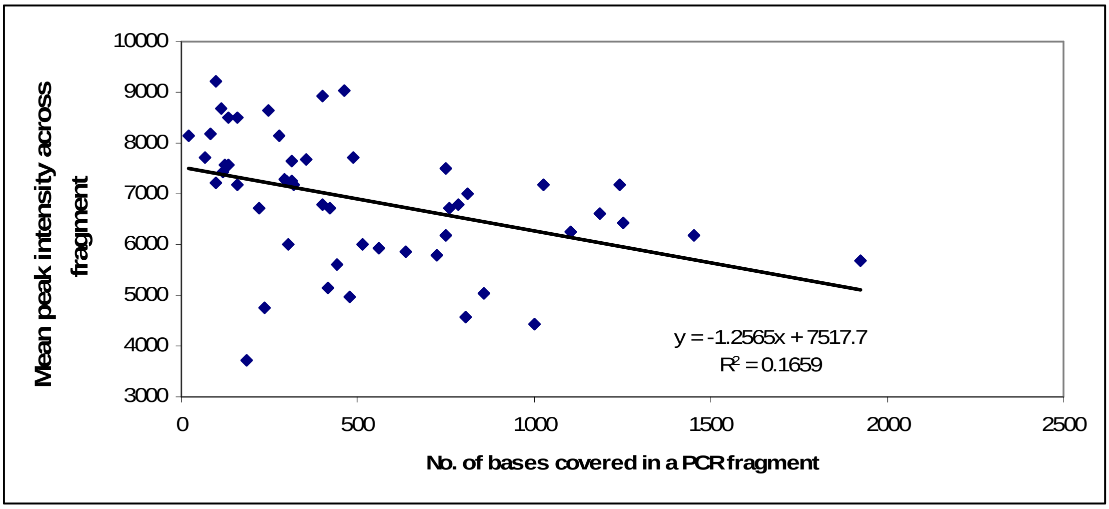

Variant detection and GC Content: For each array, variants were classified based on whether they could be identified correctly or if they were missed or not called by GSEQ. GC-content was calculated for all probes that interrogated a variant position on the array. A total of 240 variants (59 unique variants) from 13 Cincinnati arrays were used for analysis. GSEQ correctly identified 184 of these variants while 45 were not called and 2 were called wild-type. Mean and median GC-contents were (55.7%, 56%) for correctly identified variants and (64.8%, 64%) for not called and missed variants. An Anderson-Darling test on the two groups indicated a non-normal distribution, thus a non-parametric test was used instead of the two-sample t-test. The Wilcoxon rank sum test rejected the null hypothesis of equal median GC-content for the two groups with a p-value of 1e-07 at 5% significance level, indicating a statistically significant impact of GC-content on variant detection.

Supplementary Method: Proposed Approach for Resolution of No-Calls: Strand-specific Probe Cell Intensity Comparison for Filtering GDAS/GSEQ Calls (sPROFILER)

Note: MATLAB code is provided as a separate file (MatlabCode.txt).

Invention Disclosure 106-013 "Novel Algorithm and Computational Program for the Resolution of Sequence Information from Affymetrix Gene Chip Operating System Application Output."
University of Cincinnati
Intellectual Property Office
3130 Highland Ave, Third Floor
Cincinnati, Ohio 45219-2374

sPROFILER – Algorithmic Design and Implementation

sPROFILER attempts to resolve no-calls obtained from GDAS/GSEQ by looking for a distinct intensity signature on individual strands so that if one strand performs worse than the other due to sequence-specific hybridization artifacts, the base can still be called based on a clear signature from a single strand. This approach complements GDAS/GSEQ base-calling scheme which requires clear intensity signals on both strands. The process has been divided into the following stages:

- Analyze experiment data with GDAS/GSEQ.
- Stream the intensity data, reference sequence and the calls made by GDAS/GSEQ for each of the sites into sPROFILER.
- Relax the base-calling criteria and resolve ONLY the No Calls at this stage of sequence analysis. Critical algorithm settings and thresholds can be modified by the user based on the desired level of stringency.
- Assign a quality score to each resolved call. This would indicate the confidence with which the base has been called and would leave it to the investigator's discretion to accept or reject calls with relatively low quality scores.

1.1 Algorithm Design

sPROFILER analyzes every chip individually and takes the reference sequence, the final sequence obtained from GDAS/GSEQ, and the raw intensity values for all sites as input. It determines the GDAS/GSEQ call for every site by stepping through all the sites and goes through the following series of steps if a 'No Call' is encountered.

The rationale behind the algorithm is that if a site was marked as 'no call', it can possibly have a clear peak corresponding to the wild-type base on one strand but weak signal on the other strand or the site may not have a signal corresponding to wild-type base on either strand and is ruled as a 'no call' again.

1.2 Implementation

After running a Batch Analysis on GDAS and obtaining chip reports, each chip is individually analyzed with sPROFILER. The algorithm has been implemented in MATLAB. MATLAB provides a plethora of in-built functions for dealing with matrices and this leads to considerable reduction in programming complexity as well as the running time. Perl scripts have been implemented to process GSEQ data and format the CHP and CEL files to be analyzed with sPROFILER.

In order to cross-validate the results obtained from GDAS/GSEQ and the program, sequencing has been performed for the arrays. We have incorporated validation capability in the program so that it gives us all the data pertinent to its performance when compared against the "Gold Standard" which are the sequencing results. It should be kept in mind that sequencing information is not used in any way to affect the resequencing output. It is solely used for validation to determine how many true variants are missed or detected by GDAS/GSEQ and the program. This feature will be covered in more detail in a subsequent section.

1.2.1. Without cross-validation against sequencing results (used when sequencing results are not available)

The program requires input files in the form of CEL and CHP data for each chip to be analyzed. A text file containing the chip numbers for all the chips which need to be analyzed is created, each line representing one chip. All the input files should be saved in the same working directory. Associated with each chip is a CEL file and a CHP file text file with intensities for all sites on the chip. The naming convention is that for chip ChipX, the files have to be named 'ChipX_int.txt' and 'ChipX_calls.txt' to be recognized by the program.

Once the above files have been stored in the directory, the program is invoked from MATLAB using the function BaseCallMain. This function takes 1 input argument – Chips.txt. This is all the user needs to do and from thereon the program steps through the analysis of every chip in the Chips.txt file. To elaborate more on the methodology, let us consider the case of a chip, Chip1, which consists of N base pairs. Following are the different stages involved in analysis of a single chip:

1. Read the intensity values from Chip1_int.txt.
2. Read the calls from Chip1_calls.txt.
3. If >75% of neighboring calls are variant or No-calls in a +/-12-bp window, convert the base call to a no-call and do not attempt to resolve it. If Quality Score for a variant call is <55, convert it to No-call (false positive filters adapted from Pandya et al).
4. We use the information available from GDAS/GSEQ to set the thresholds for making heterozygous calls. The algorithm steps through all the nucleotide positions, excludes the ones with no-calls and variant calls from GDAS/GSEQ, and calculates the following four ratios:

- peak43F: It is the mean ratio, taken over all wild-type calls made by GDAS/GSEQ, for the peak intensity and the next highest intensity.
- peak32F: It is the mean ratio, taken over all wild-type calls made by GDAS/GSEQ, for the 2nd highest and 3rd highest intensity values.
- peak43R: Anti-sense counterpart of peak43F.
- peak32R: Anti-sense counterpart of peak32F.

5. **Check if ONE of the strands shows a clear peak AND corresponds to the refseq**: The site is tested to see if the following criteria are met:
   - Maximum intensity on sense or anti-sense matches the base on refseq.
   - The maximum value exceeds the next highest by a certain factor (see the next section on comparison of threshold ratios).
   - The site is called based on the peak intensity if these conditions are met.

   - Assign a quality score based on the following formula:

$$\text{Score} = \frac{\text{Highest(fwd)*Highest(rev)* Highest(fwd)*Highest(rev)}}{\text{2ndHigh(fwd)*2ndHigh(rev)*meanLow3(fwd)*meanLow3(rev)}}$$

6. If none of the above set of conditions is met, assign a ‘No Call’ to the site.
7. If the site was being called by GDAS/GSEQ, retain that call.
8. Assign a flag to each call as follows. This would help the user in filtering the calls while analyzing the data.
   - If the call from GDAS/GSEQ matches refseq, Flag=0;
   - If no-call is converted to refseq, Flag=1;
   - If GDAS/GSEQ detected a Het on the site, Flag=2;
   - If GDAS/GSEQ detected a homozygous variant on the site, Flag=3;
   - If the site is still a ‘No Call’, Flag =4;

9. Print results for each chip in the file ‘ChipNum_final.txt’. It comprises of the GDAS calls, refseq, calls made by the program, Quality score, Model type, Call Type and the 8 intensities. Figure 2 illustrates a sample of the output file.

Oto07_final - Notepad

File Edit Format View Help

Index	New	Ref	GDAS	Score	Model	Flag	A	C	G	T	A"	C"	G"	T"
1	a	a	a	0	0	0	5335.1	2916.6	6932.5	12682.9	10978.8	3148.4	2242.1	3479.4
2	c	c	c	0	0	0	3105.3	3286.4	13848.7	4318.5	4401.2	11848.2	2601.9	2588.5
3	a	a	a	0	0	0	6412	5901.8	6126.2	12319.8	11071.3	3508.9	4985	6532.5
4	g	g	g	0	0	0	6478.4	12241.5	4713.2	4674.3	3161.9	2482.6	11200.3	2382.4
5	c	c	c	0	0	0	3588.9	3724.6	12072.9	2771.6	4738.4	11851.6	3078.3	4269.1
6	g	g	g	0	0	0	3074.4	10059.2	2657	2789.7	2828.7	2751.6	11367.5	2191.5
7	a	a	a	0	0	0	2465.6	2619.3	2230.5	8246.7	11369	2036.4	2958.3	4166.8
8	g	g	g	0	0	0	1709.6	7093.8	1403.7	1012.5	1755.5	1452.4	11226.9	1580.8
9	t	t	t	0	0	0	6755.5	844.2	1075.8	1326.6	2530.7	1640	4242.6	11290.9
10	g	g	g	0	0	0	2504.6	7752.7	2239.7	1776.3	2700.2	3459	13306.3	2855.4
11	c	c	c	0	0	0	1702.5	1768	7987.9	1478.5	6508.3	12822.6	5084.1	3505.4
12	t	t	t	0	0	0	8247.1	2352	3148.5	4443.1	6281.6	5865.9	7450.8	13659.9
13	a	a	a	0	0	0	3391.5	2917.7	4835.6	9129.6	14744.2	5864.7	7293.6	6435.8
14	c	c	c	0	0	0	3354.7	2564.4	10292.6	3165.7	8632.5	16416.7	6886.9	7588.5
15	g	g	g	0	0	0	3969	9451.5	1729.4	3895.6	5557.5	6541.9	15337.5	3871.7
16	c	c	c	0	0	0	4508.2	5012.6	10150.9	3523.3	10384.3	15306.7	9077.7	7385.8
17	c	c	c	0	0	0	2905.8	1993.2	9379	2049.2	7281.5	13628.6	5127.3	5697
18	t	t	n	45.0825	2	0	14584.2	2757.3	3061.6	4728.3	6785.5	5515	7454	13150.7
19	a	a	a	0	0	0	3434.5	2367.7	3611	10288.8	12925.6	3589	5408.9	5631.1
20	a	a	a	0	0	0	2712.3	1440.9	2794.9	10614.6	13021.3	3057.4	3319.8	4328.4
21	a	a	a	0	0	0	3347.5	2592.5	4447	10465.4	12253.4	3269.8	3539.4	3364.1
22	c	c	c	0	0	0	1534.6	1193.8	10834.3	1281.5	4389.7	12039	3222.7	2618.6
23	t	t	t	0	0	0	10064.3	1773.7	2362.9	3143.1	2605.7	3072	3647	10746.8
24	g	g	g	0	0	0	4319.1	10137.6	2657.4	2673.1	2664.8	2898.8	11260	2833
25	g	g	g	0	0	0	4569	9855.1	3570.2	3004.5	3594.4	3954.9	11146.9	2782.2
26	c	c	c	0	0	0	1950.2	1928.9	10214.7	1465.1	6065.7	11829.9	4062.4	3867.2
27	t	t	t	0	0	0	10290.6	2431	2937.2	3399.6	3697.3	3670.2	5051.4	10780.2
28	g	g	g	0	0	0	1989.7	9178.5	1911.4	2057.4	2092.8	2028	10981.7	1748.1
29	t	t	n	20.8693	2	0	9549.5	2972.9	3702	2718	4482.3	3649.7	7117.5	9796.3
30	c	c	c	0	0	0	1854.1	1670.6	9906.1	1526.1	4266.4	9847.6	2706.4	2209.7

Figure 2: ChipNum_final.txt - Sample Output File with Results for a Single Chip

10. Print all the variants, those obtained from GDAS/GSEQ and the ones from the new program, for each chip in 'ChipNum_OutSnps.txt.'

585B_OutSnps - Notepad

File Edit Format View Help

Index	Ref	New	GDAS	Score	Model	Flag	A	C	G	T	A"	C"	G"	T"
957	t	w	w	0	0	2	15264.2	10310	11165.5	12618.1	3327.7	2154.9	2004.4	4159.1
1851	c	m	m	0	0	2	301	246.6	513.8	408.8	6398.3	5966.9	2222.8	3921.7
1855	c	t	t	0	0	3	849	566.6	514.3	703.1	4338.9	4791.7	3055.5	4492.1
1924	a	w	w	0	0	2	14247.9	7306.6	6772.6	9737	740.9	517.1	502	758.1
2065	g	r	r	0	0	2	2126.4	4283.1	1603.5	3994.6	8798.3	5629.1	8396	5116.6
2322	c	s	s	0	0	2	2521.8	4579.7	5001.3	2722.7	7008.8	11051	8698	4883.7
2465	g	k	k	0	0	2	6011.4	7708.9	3151.5	3307.6	4290.6	3994.1	4376.4	4834.5
2772	g	r	r	0	0	2	1381.6	3991.1	1302.6	4381.9	8674.3	4720.6	10037.8	6153.9
2830	g	c	c	0	0	3	4748.1	5535.7	5668.5	5257.2	4261.4	8133.3	3054.6	3148.2
2846	c	t	t	0	0	3	6780.3	2128.4	2023.1	2821.4	2455.6	3313.4	3739.5	7151
3537	g	r	r	0	0	2	186.7	303.4	247.7	198.7	3842.4	1880.8	3391	1405.1
3551	c	a	a	0	0	3	180.5	172.7	213.8	224.1	4786.3	2808.8	1892.1	2648.8
3570	g	s	s	0	0	2	309.8	474.7	503.7	235.6	1581.1	2729.3	2837.8	1942
3578	t	w	w	0	0	2	242	197.5	191.1	230.1	4179.8	3551	2209.1	4453.5
3587	c	m	m	0	0	2	233.9	207.8	270.9	322.6	2095	2343.2	1067.2	966.5

Figure 3: ChipNum_OutSnps.txt - Sample Output File with all the Variants for a Single Chip

1.2.2. With cross-validation against sequencing results (used when sequencing results are available)

- When sequencing results are available for a chip, the program can be used to validate the output against sequencing data. The user needs to create a file, 'ChipNum_knownSnp.txt' which comprises of all the locations at which a variant was found with sequencing along with the actual base called at that location. An example is shown below:

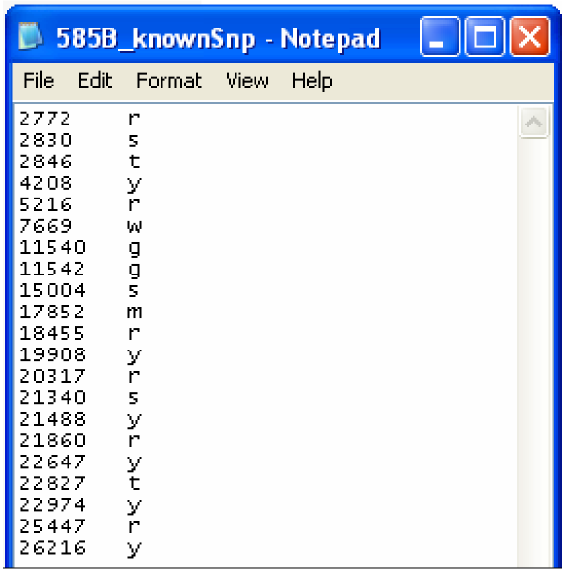

2772	r
2830	s
2846	t
4208	y
5216	r
7669	w
11540	g
11542	g
15004	s
17852	m
18455	r
19908	y
20317	r
21340	s
21488	y
21860	r
22647	y
22827	t
22974	y
25447	r
26216	y

Figure 4: ChipNum_knownSnp.txt – Input file with Known Snp Locations and Corresponding Calls

- Once this file has been created, all one needs to do to include a cross-validation is to save this file in the corresponding chip folder. For example, the above file will be saved in the folder /585B. On seeing this file in the folder, the algorithm detects that sequencing results are available and that cross-validation has to be performed.
- All the steps remain the same as in the case where we do not have sequencing data available. However, additional information is generated which indicates how many true variants are missed or detected in the final resequencing output.
- In 'ChipNum_OutKnownSnp.txt.', all the known variant locations are listed along with the call obtained from sequencing, GDAS/GSEQ and sPROFILER. It also includes the model type and corresponding scores. A sample is shown below:

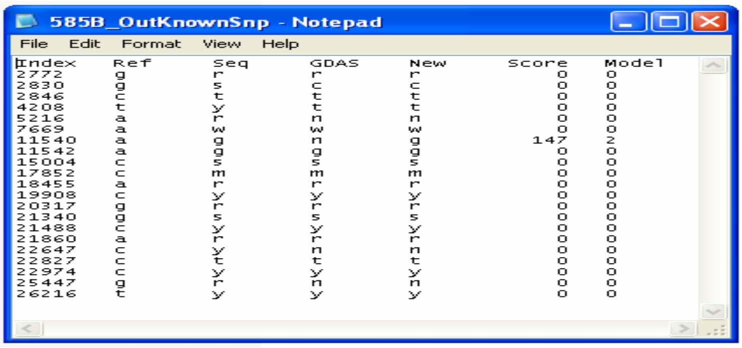

Index	Ref	Seq	GDAS	New	Score	Model
2772	g	r	r	r	0	0
2830	g	s	c	c	0	0
2846	c	t	t	t	0	0
4208	t	y	t	t	0	0
5216	a	r	n	n	0	0
7669	a	w	w	w	0	0
11540	a	g	n	g	147	2
11542	a	g	g	g	0	0
15004	c	s	s	s	0	0
17852	c	m	m	m	0	0
18455	a	r	r	r	0	0
19908	c	y	y	y	0	0
20317	g	r	r	r	0	0
21340	g	s	s	s	0	0
21488	c	y	y	y	0	0
21860	a	r	r	r	0	0
22647	c	y	n	n	0	0
22827	c	t	t	t	0	0
22974	c	y	y	y	0	0
25447	g	r	n	n	0	0
26216	t	y	y	y	0	0

Figure 5: ChipNum_OutKnownSnp.txt – Output file Containing Calls Made by Different Programs for Positions known to have Variants

Generation of final output: A Perl script processes sPROFILER calls and generates an output with remnant no-calls and variant calls along with attributes such as corresponding GC content, coding position, etc, as shown below.

Names	Description	Examples
AffyID	Affymetrix base ID – 1 through 27106	1650
Frag	Reference fragment and the base position	CDH23_ex46_68*-*12*
Refseq	Reference base	c
CDS	Coding position of the base	23
ChipName_sPROFILER	All variant calls and no-calls that remain after patch application to GSEQ calls	n
ChipName_GSEQ	GSEQ calls corresponding to the no-calls and variant calls in column E that remain after application of sPROFILER. Note: Some of the GSEQ variant calls within N-stretches or with low quality scores are converted to no-calls with the patch	c
ChipName _Qual	Quality score	120.399
ChipName _exonCallRate	Exon call rate (%)	98.9
GC	Probe GC content (%)	48
NSeq	nearby sequence with +/-2 flanking bases	ct**c**tc
NCalls	Total number of samples without calls at this position (out of all the samples included in the analysis)	2
NChips	Samples without calls at this position	010, 030
NumWT	Number of sample out of total samples for which dideoxy sequencing showed wild-type calls	13_13
FPCalls	Total number of samples with False Positive Calls	2
FPChips	Samples with False Positive Calls	023, 241
BaseExCallRate	Average exon call rate for baseline arrays (%)	99.4
VarOrN	Variant or no-call in the analyzed chip	N
Unique	Unique co-calls	UniqueN

Comparison of methods for calculation of site-specific thresholds for making wild-type calls with sPROFILER: We evaluated five different methods for determining site-specific peak to next highest intensity ratios for making wild-type calls with sPROFILER, namely 1) average threshold ratio obtained from using all wild-type calls from GSEQ within the same array (Within-array) 2) average threshold ratio obtained from all wild-type calls on the site of interest across all arrays (Across-arrays; Exception: if no wild-type call was made at a particular position across arrays, Within-array ratio was used if the site was not called wild-type in any of the arrays) 3) combination of Within-array ratio and a scaling factor, obtained from the proportion of base calls (exclude no-calls) for the site that were called wild-type by GSEQ across all arrays 4) combination of Across-arrays and the scaling factor, and 5) combination of Within- and Across-arrays. Table 5 presents results from comparing different schemes for calculation of site-specific threshold ratios for making wild-type calls. We used the 13 optimized Cincinnati arrays for the analysis. GSEQ provided highest sensitivity but lowest specificity and call coverage. Across all methods, highest sensitivity was obtained when the threshold was calculated by using average peak to next highest intensity ratio across all wild-type calls within the same array and scaling it with the proportion of base calls at

the site that were called wild-type across all arrays (Within-array + Scaling in Table 5). The remaining methods provided higher specificity and fewer no-calls but lower sensitivity. We chose the most conservative method (Within-array+Scaling) for our analysis in order to minimize the increase in number of false negatives while reducing no-calls and false positives.

Table 5: Comparison of schemes for calculation of site-specific threshold for peak to next highest intensity ratio

	No. of no-calls*	FP*	FN*	Sensitivity	Specificity
**GSEQ**	7317 (563)	549 (42)	6 (0.46)	96.92	97.77
**Within-array**	810 (62)	398 (30)	15 (1.15)	93.04	99.66
**Across-arrays**	738 (57)	398 (30)	17 (1.30)	92.44	99.68
**Within-array+Scaling**	1338 (103)	398 (30)	11 (0.85)	94.61	99.51
**Across-arrays+Scaling**	745 (57)	398 (30)	17 (1.30)	92.44	99.68
**Within-array+Across-arrays**	456 (35)	398 (30)	17 (1.30)	92.44	99.76

***values represented as Total bases and (per chip average).**
